# Nuclear Parkin Activates the ERRα Transcriptional Program and Drives Widespread Changes in Gene Expression Following Hypoxia

**DOI:** 10.1038/s41598-020-65438-7

**Published:** 2020-05-22

**Authors:** Sarah E. Shires, Justin M. Quiles, Rita H. Najor, Leonardo J. Leon, Melissa Q. Cortez, Mark A. Lampert, Adam Mark, Åsa B. Gustafsson

**Affiliations:** 1 Skaggs School of Pharmacy and Pharmaceutical Sciences, Department of Pharmacology, University of California, San Diego, La Jolla, CA USA; 2 Center for Computational Biology & Bioinformatics, Department of Medicine, University of California, San Diego, La Jolla, CA USA

**Keywords:** Cell biology, Molecular biology, Cardiology, Diseases

## Abstract

Parkin is an E3 ubiquitin ligase well-known for facilitating clearance of damaged mitochondria by ubiquitinating proteins on the outer mitochondrial membrane. However, knowledge of Parkin’s functions beyond mitophagy is still limited. Here, we demonstrate that Parkin has functions in the nucleus and that Parkinson’s disease-associated Parkin mutants, ParkinR42P and ParkinG430D, are selectively excluded from the nucleus. Further, Parkin translocates to the nucleus in response to hypoxia which correlates with increased ubiquitination of nuclear proteins. The serine-threonine kinase PINK1 is responsible for recruiting Parkin to mitochondria, but translocation of Parkin to the nucleus occurs independently of PINK1. Transcriptomic analyses of HeLa cells overexpressing wild type or a nuclear-targeted Parkin revealed that during hypoxia, Parkin contributes to both increased and decreased transcription of genes involved in regulating multiple metabolic pathways. Furthermore, a proteomics screen comparing ubiquitinated proteins in hearts from Parkin^−/−^ and Parkin transgenic mice identified the transcription factor estrogen-related receptor α (ERRα) as a potential Parkin target. Co-immunoprecipitation confirmed that nuclear-targeted Parkin interacts with and ubiquitinates ERRα. Further analysis uncovered that nuclear Parkin increases the transcriptional activity of ERRα. Overall, our study supports diverse roles for Parkin and demonstrates that nuclear Parkin regulates transcription of genes involved in multiple metabolic pathways.

## Introduction

Parkin is an E3 ubiquitin ligase primarily known for its role in facilitating the selective elimination of dysfunctional mitochondria through a process called mitochondrial autophagy (mitophagy). Upon loss of mitochondrial membrane potential, Parkin is rapidly recruited to the outer mitochondrial membrane by the serine/threonine kinase PINK1 to ubiquitinate a host of proteins on the outer mitochondrial membrane which flags the damaged mitochondrion for sequestration and degradation through autophagy^1,2^. Parkin-deficiency is associated with development of Parkinson’s disease (PD)^3^ and skeletal muscle atrophy^4^. Parkin also facilitates cardiac metabolic maturation in the neonatal heart and adaptation to acute cardiac stress such as myocardial infarction^5,6^. Additionally, Parkin functions as a potential tumor suppressor and is mutated in several human cancers^7^. As such, Parkin plays a critical role in maintaining health in a variety of cells and tissues.

Parkin is named for its link to autosomal recessive Parkinson’s disease (PD)^8,9^. Although loss of function mutations in Parkin contribute to loss of dopaminergic neurons, the underlying mechanisms that precipitate the neuronal degeneration associated with PD are still not fully understood. Mitochondrial impairment is a consistent feature in PD and initial studies in *Drosophila melanogaster* confirmed that loss of Parkin leads to widespread mitochondrial dysfunction and muscle degeneration^10^. Based mostly on *in vitro* studies, the pathogenic phenotypes observed in Parkin-deficient cells and tissues have generally been attributed to its role in mitophagy. However, emerging evidence suggests that Parkin’s functions extend beyond mitophagy and it is unlikely that mitophagy defects are solely responsible for the pathological phenotypes associated with Parkin-deficiency.

As a cytosolic E3 ubiquitin ligase, Parkin has the capability of regulating numerous cellular processes through diverse protein substrates. For example, Parkin can activate mitochondrial biogenesis by ubiquitinating and promoting degradation of cytosolic PARIS, a repressor of peroxisome proliferator-activated receptor gamma coactivator 1α (PGC-1α)^11^. Additionally, Parkin can regulate lipid metabolism by stabilizing the plasma membrane CD36 lipid transporter, which causes Parkin-deficient mice on high-fat diet (HFD) to withstand weight gain, steatohepatitis, and insulin resistance^12^. Parkin can also directly inhibit apoptosis by ubiquitinating pro-apoptotic Bax, thus prohibiting its translocation from the cytosol to mitochondria in response to apoptotic stimuli^1,13^. More recently, Parkin was shown to negatively regulate inflammation via inhibition of RIPK3, an initiator of necroptosis^14^. Overall, these studies demonstrate that Parkin is a complex protein with multiple functions that contribute to cellular homeostasis and survival.

To date, most investigations have focused on understanding Parkin’s role in mitophagy and its link to mitochondrial dysfunction and cell death. Therefore, our knowledge of Parkin’s functions beyond mitophagy is still very limited. Here, we establish that Parkin is recruited to the nucleus during hypoxia where it mediates changes in gene transcription. Our findings also demonstrate that nuclear Parkin interacts with the transcription factor ERRα to enhance its transcriptional activity.

## Results

### Parkin is detected in both cytosolic and nuclear fractions *in vitro* and *in vivo*

**Figure 1:**
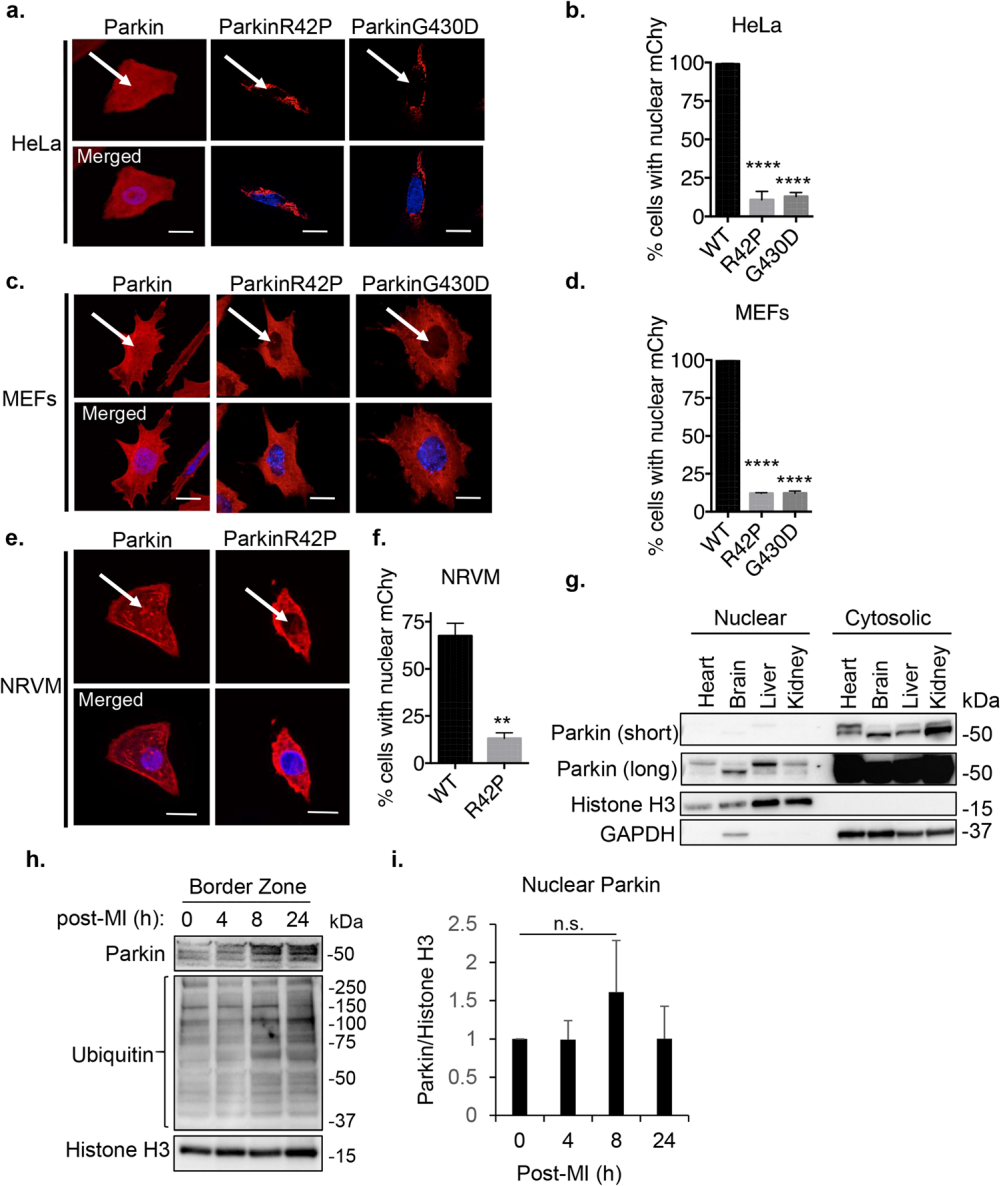
Parkin localizes to the nucleus *in vitro* and *in vivo*. (**a**) Representative fluorescence images of HeLa cells expressing mCherry-Parkin, mCherry-ParkinR42P, or mCherry-ParkinG430D. Scale bar = 20 µm. (**b**) Quantification of the number of HeLa cells with Parkin present in the nucleus. A minimum of 100 cells were counted for n = 3. (**c**) Representative fluorescence images of MEFs expressing mCherry-Parkin, mCherry-ParkinR42P, or mCherry-ParkinG430D. Scale bar = 20 µm. (**d**) Quantification of the number of MEFs with Parkin present in the nucleus. A minimum of 100 cells were counted for n = 3. (**e**) Representative fluorescence images of NRVMs expressing mCherry-Parkin or mCherry-ParkinR42P. Scale bar = 10 µm. (**f**) Quantification of the number of NRVMs with Parkin present in the nucleus. A minimum of 40 cells were counted for n = 3. (**g**) Western blot analysis of nuclear and cytosolic fractions prepared from mouse tissues. Histone H3 and GAPDH were used as loading controls. (**h**) Representative Western blot analysis of nuclear fractions prepared from myocardial infarct border zone. Histone H3 was used as loading controls. (**i**) Quantification of Parkin levels in the nuclear fractions (n = 4). **p < 0.01, ****p < 0.0001 compared to WT Parkin, n.s. = not significant. Full length blots are available in Supplementary Figure S6.

To examine the cellular localization of Parkin, we overexpressed mCherry-tagged Parkin in various cell types and analyzed its localization by fluorescence microscopy. As previously reported, Parkin was distributed throughout the cell at baseline in HeLa cells, mouse embryonic fibroblasts (MEFs) and neonatal rat ventricular myocytes (NRVMs) (Fig. 1a-f, Suppl Fig. S1). Unexpectedly, two different Parkinson’s disease-associated mutants of Parkin, ParkinR42P and ParkinG430D, were selectively excluded from the nucleus in all three cell types tested. Due to very low expression level of ParkinG430D in NRVMs, we were unable to assess the cellular localization of this mutant in these cells. The reason for the low expression in the NRVMs is currently unclear. Next, we examined the subcellular localization of endogenous Parkin in tissues that express high levels of Parkin. To confirm the nuclear localization of endogenous Parkin, we performed Western blotting of nuclear and cytosolic fractionations prepared from different mouse tissues. Consistent with previous studies, Parkin was predominantly present in the cytosolic fractions of mouse heart, brain, liver and kidney (Fig. 1g). However, we noted that a portion of Parkin was also detected in the nuclear fractions of these tissues. We have previously reported that Parkin is activated in the border zone tissue of the infarcted heart^5^. Therefore, we examined if endogenous Parkin also translocated to the nucleus under these conditions. We found that Parkin levels tended to increase in the nuclear fraction prepared from the infarct border zone tissue after permanent ligation of the left anterior descending coronary artery (Fig. 1h). The increase in Parkin in nucleus did not reach statistical significance due to the variability between experiments (Fig. 1i). These results suggest that functional Parkin localizes to the nucleus in cells and translocates to the nucleus in response to pathophysiological stressors.

**Figure 2:**
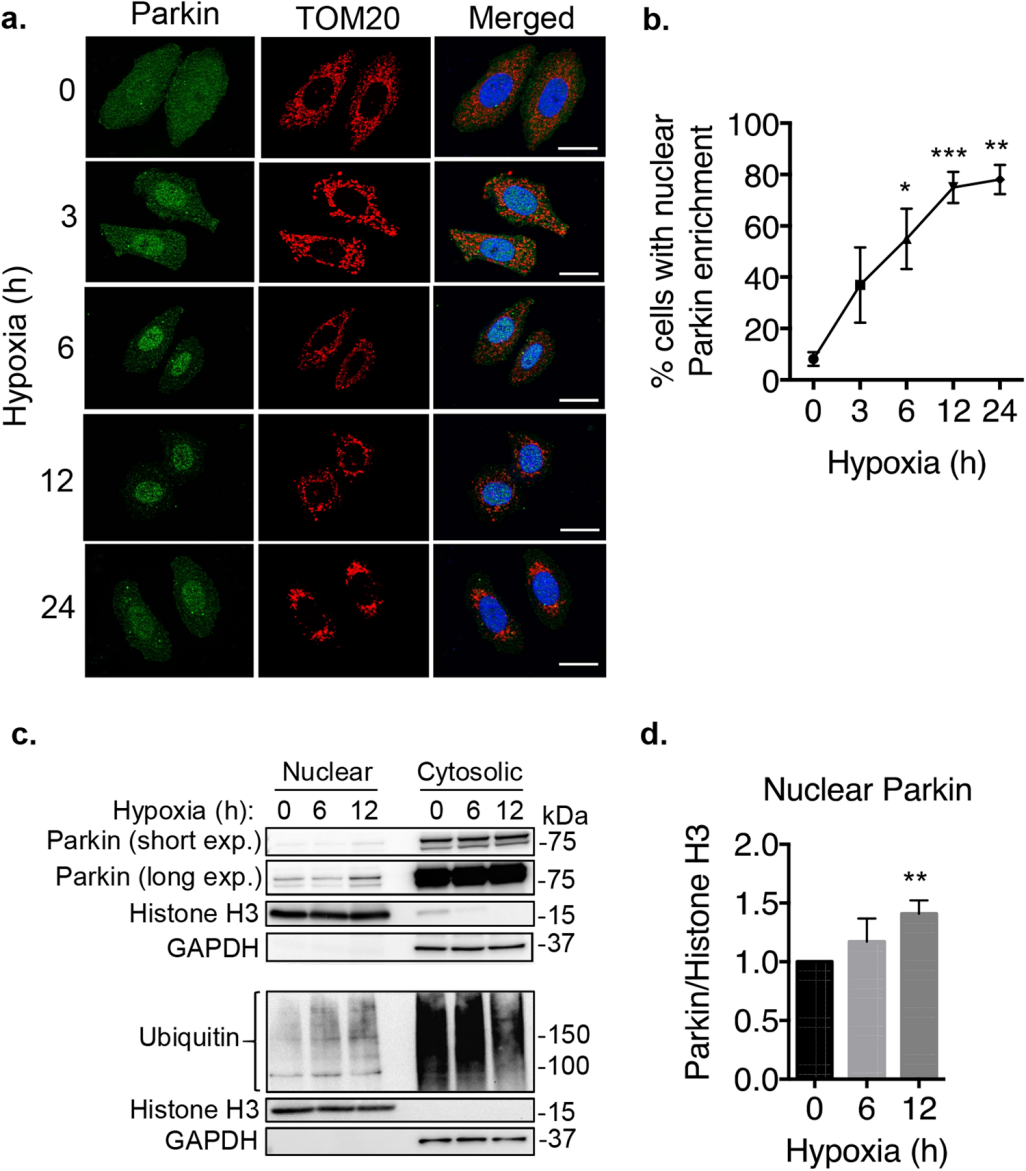
Parkin translocates to the nucleus during hypoxia. (**a**) Representative fluorescence images of YFP-Parkin and TOM20 in HeLa cells subjected to hypoxia. (**b**) Quantification of the number of HeLa cells with nuclear Parkin enrichment. A minimum of 100 cells were counted per condition in three independent experiments (n = 3). (**c**) Representative Western blots of nuclear and cytosolic fractions prepared from HeLa cells expressing mCherry-Parkin. Histone H3 and GAPDH were used as loading controls. (**d**) Quantification of Parkin levels in the nuclear fraction of HeLa cells (n = 5). Scale bars = 20 µm. *p < 0.05, **p < 0.01, ***p < 0.001 compared to 0 hr. Full length blots are available in Supplementary Figure S6.

To induce mitophagy, Parkin translocates from the cytosol to damaged mitochondria where it ubiquitinates various substrates on the outer mitochondrial membrane^15^. To assess the dynamics of Parkin’s nuclear localization, we subjected HeLa cells stably expressing YFP-Parkin to hypoxia and monitored the change in Parkin’s localization. A time-course experiment demonstrated that levels of YFP-Parkin increased in the nucleus in response to hypoxia (Fig. 2a,b). The immunofluorescence analysis of Parkin’s localization also confirmed its selective enrichment in the nucleus and not at the mitochondria during hypoxia (Fig. 2a,b). The increase in nuclear Parkin during hypoxia was also confirmed by Western blotting of nuclear fractions prepared from HeLa cells at the time points indicated (Fig. 2c,d). While immunostaining revealed significant accumulation of nuclear Parkin following hypoxia, Western blotting of cytosolic fractions showed that most of Parkin remained in the cytosol during hypoxia. This inconsistency in Parkin localization between fluorescence imaging and Western blotting experiments has previously been observed in studies on Parkin and its translocation to depolarized mitochondria^2,16,17^. Nevertheless, consistent with its role as an E3 ligase, increased nuclear Parkin also correlated with an increase in ubiquitination of nuclear proteins (Fig. 2c).

**Figure 3:**
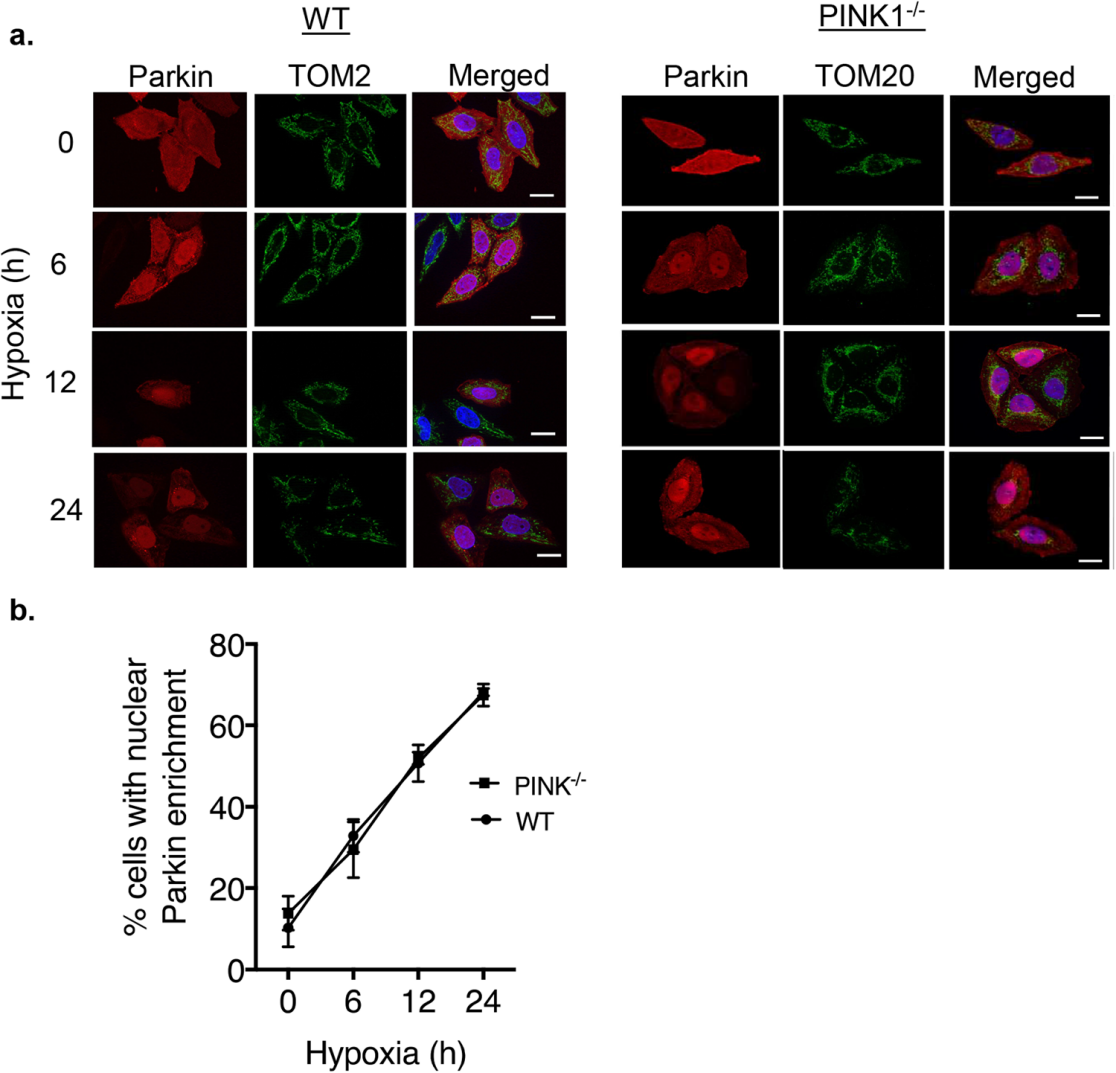
Hypoxia-induced Parkin translocation to the nucleus is independent of PINK1. (**a**) Representative fluorescence images of mCherry-Parkin and TOM20 in WT (left) and PINK1^−/−^ HeLa cells (right). (**b**) Quantification of the number of WT and PINK1^−/−^ HeLa cells with nuclear Parkin enrichment. A minimum of 100 cells counted per condition in three independent experiments (n = 3). Scale bars = 20 µm.

The serine/threonine kinase PINK1 is a known upstream regulator of Parkin-mediated mitophagy and is required for Parkin’s translocation to mitochondria^18–20^. Although PINK1 is mainly known as the upstream regulator of Parkin during mitophagy, there are reports that PINK1 also has functions in the cytosol^21–23^. Therefore, we investigated whether PINK1 is required for Parkin’s recruitment to the nucleus by monitoring Parkin’s translocation to the nucleus in PINK1-deficient HeLa cells^24^. We found that nuclear Parkin levels were increased to a similar extent in both WT and PINK1-deficient HeLa cells during hypoxia (Fig. 3a,b), suggesting that its translocation to the nucleus during hypoxia occurs independently of PINK1. Finally, we examined the effect of nutrient deprivation on Parkin’s subcellular localization. In contrast to hypoxia, incubating HeLa cells in media lacking serum and glucose for up to one hour led to rapid efflux of Parkin from the nucleus. Immunofluorescence and Western blotting of nuclear fractions demonstrated that Parkin exited the nucleus after just 15 min of nutrient deprivation (Suppl Fig. S2a-d). Taken together, these results suggest that Parkin’s localization is dynamic and that hypoxia induces the selective translocation of Parkin to the nucleus independent of PINK1.

### Nuclear Parkin alters the transcriptome in HeLa cells

**Figure 4:**
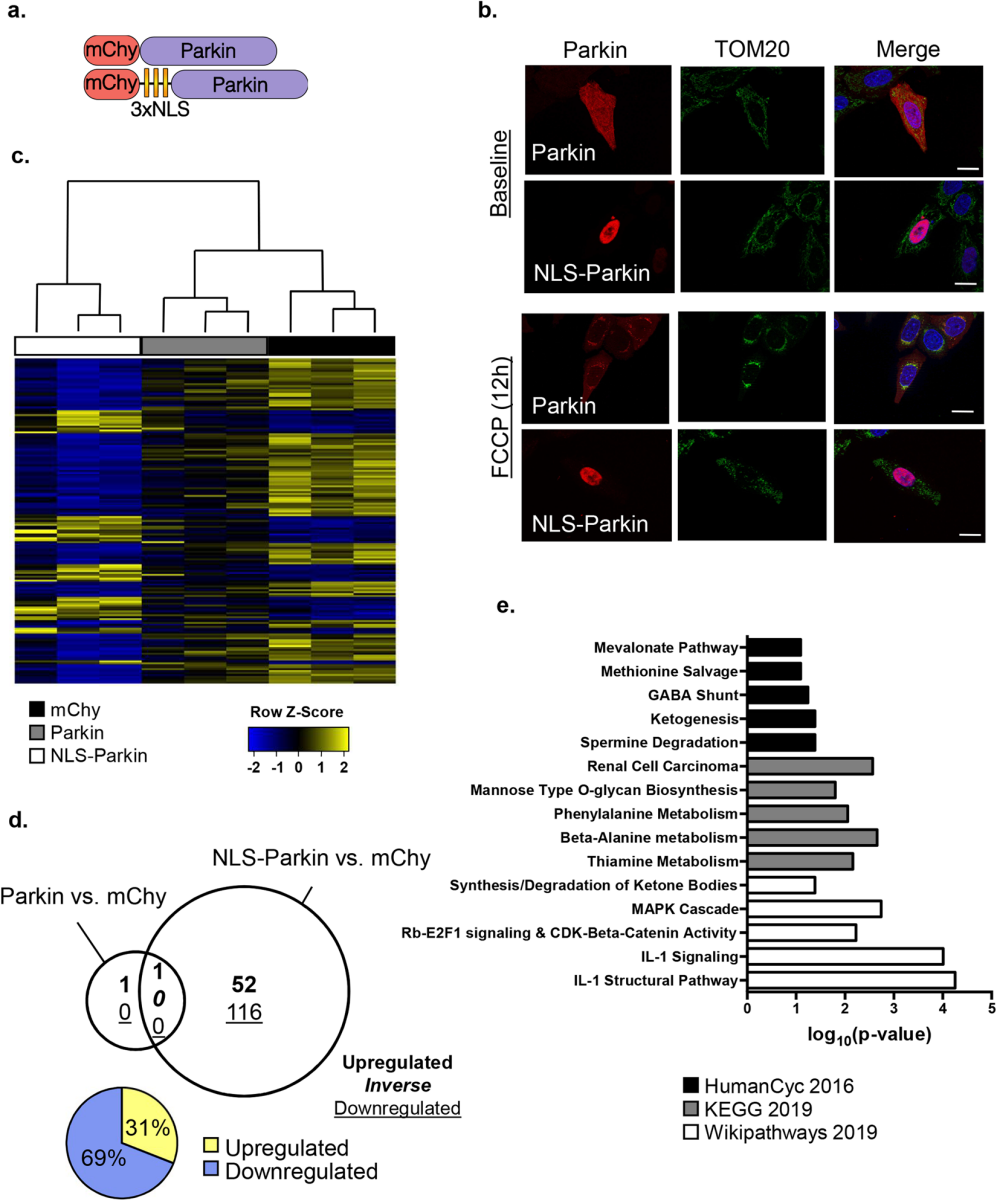
Effect of nuclear Parkin on the transcriptome under baseline conditions. (**a**) Diagram of nuclear-targeted Parkin construct. (**b**) Representative fluorescence images of HeLa cells expressing Parkin or NLS-Parkin at baseline (top) and after treatment with 25 μM FCCP for 12 h (bottom). Scale bars = 20 µm. (**c**) Hierarchical clustering heat map of all differentially expressed genes (DEGs; fold-change ≥ 1.5, FDR < 0.05) identified by RNA sequencing (RNA-seq). (**d**) Area-proportional Venn diagram of the number of DEGs. HeLa cells expressing Parkin or NLS-Parkin were compared to cells expressing mCherry. Number of upregulated genes in bold, number of downregulated genes underlined, with percentages of upregulated and downregulated genes depicted in pie chart (bottom left). (**e**) Top five biological processes determined by Gene Ontology analysis of all DEGs using HumanCyc 2016 (top), KEGG 2019 (middle) and Wikipathways 2019 (bottom).

To investigate the function of Parkin in the nucleus, we generated a nuclear-targeted Parkin by inserting three consecutive nuclear localization signals (NLS) at the N-terminus of Parkin (Fig. 4a). While Parkin localized throughout the cell under baseline conditions when overexpressed in HeLa cells and MEFs, NLS-Parkin was restricted to the nucleus (Fig. 4b & Suppl Fig. S3). Treatment of cells with mitochondrial uncouplers, such as FCCP, leads to translocation of Parkin to mitochondria^15^. To confirm that NLS-Parkin remained in the nucleus during stress, we exposed HeLa cells and MEFs overexpressing Parkin or NLS-Parkin to FCCP. While Parkin translocated to the mitochondria following the FCCP treatment, NLS-Parkin remained in the nucleus (Fig. 4b & Suppl Fig. S3).

To examine the function of nuclear-localized Parkin, we performed next-generation RNA sequencing (RNA-seq) on HeLa cells expressing mCherry, Parkin, or NLS-Parkin under baseline and hypoxic conditions. Hierarchal clustering of differentially expressed genes (DEGs; fold-change ≥ 1.5, FDR < 0.05) revealed no effect of wild type Parkin on the basal transcriptome; however, NLS-Parkin significantly altered the expression of 168 genes during normoxic conditions (Fig. 4c,d). Of the differentially expressed genes (DEGs), 116 (69%) were downregulated, while 52 (31%) were increased as a result of NLS-Parkin (Fig. 4d). Next, we employed EnrichR^25^ to evaluate the biological pathways implicated by NLS-Parkin-mediated DEGs. Using three different human pathway analysis tools within the EnrichR platform, the five most significantly enriched pathways associated with the NLS-Parkin transcriptome were ranked. Several of these pathways reflected amino acid and ketone metabolism suggesting a potential bioenergetic role for NLS-Parkin-mediated transcription (Fig. 4e). Indeed, we noted multiple nuclear receptors (PPARG, PPARA, ESR1) and co-activators (NCOA1) as potential upstream regulators of a distinct subset of metabolic transcripts among the NLS-Parkin DEGs (Suppl Fig. S4). Taken together, these data demonstrate that targeting Parkin to the nucleus affects transcription of genes involved in multiple metabolic pathways.

**Figure 5:**
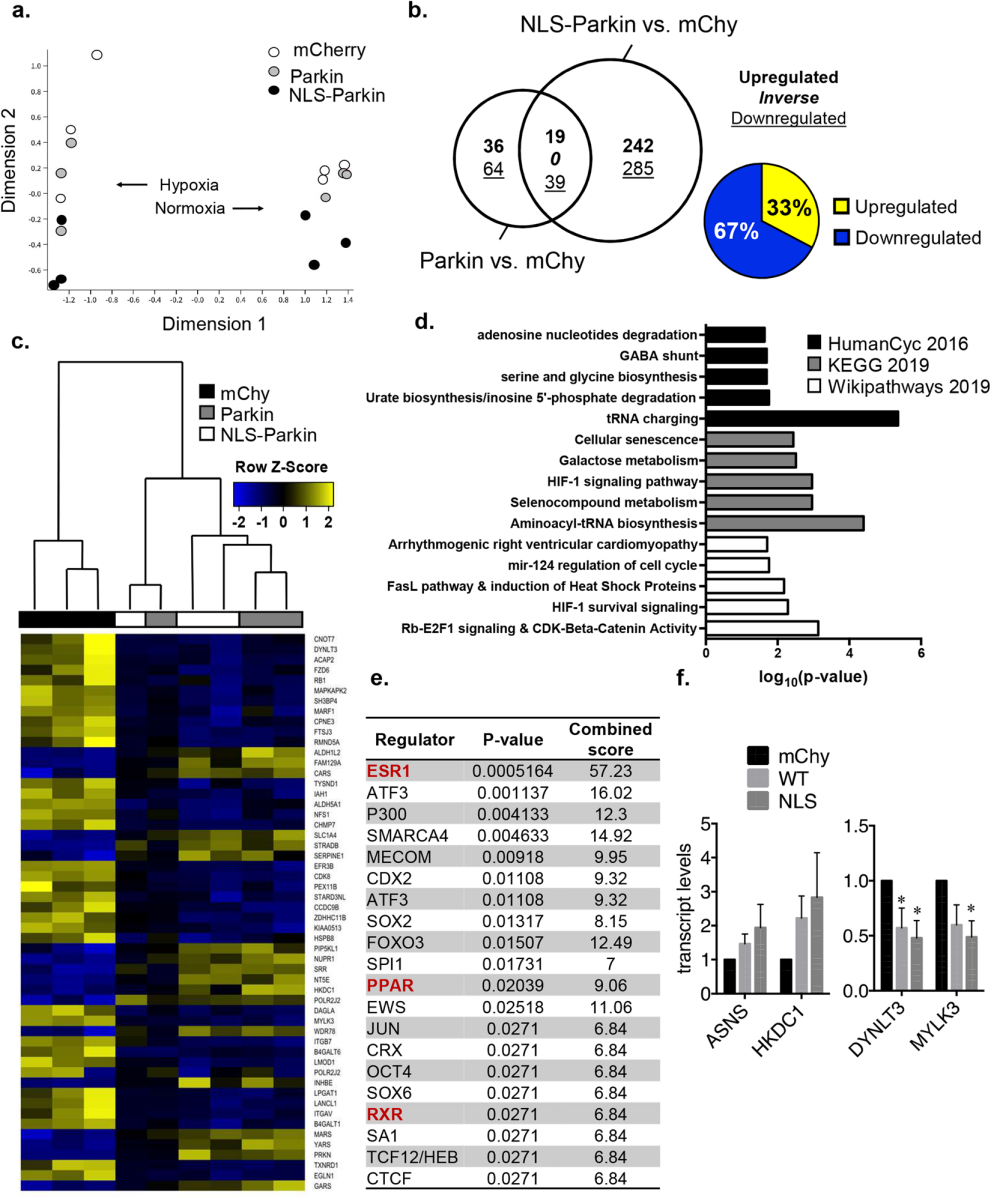
Effect of nuclear Parkin on the transcriptome in response to hypoxia. (**a**) Multi-dimensional scale (MDS) plot of samples analyzed by RNA sequencing: HeLa cells expressing either mCherry, Parkin, or NLS-Parkin at normoxia or after 12 h of hypoxia. (**b**) Area-proportional Venn diagram of the number of genes differentially expressed in response to 12 h of hypoxia comparing HeLa cells expressing Parkin or NLS-Parkin to mCherry-expressing cells. Number of upregulated genes are in bold, while number of downregulated genes are underlined. Percentages of upregulated and downregulated genes within the overlapping DEG subset depicted in a pie chart (right). (**c**) Hierarchical clustering heat map of hypoxia-responsive shared DEGs among Parkin and NLS-Parkin. (**d**) Top five biological processes determined by Gene Ontology (GO) analysis of the shared DEGs using HumanCyc 2016 (top), KEGG 2019 (middle) and Wikipathways 2019 (bottom). (**e**) EnrichR output from ChEA showing the top 20 most significantly enriched transcriptional regulators associated with Parkin and NLS-Parkin shared DEGs, nuclear receptors and cofactors emphasized in bold, red text. (**f**) qPCR validation of 4 DEGs, 2 upregulated and 2 downregulated. *p < 0.05 compared to mCherry (n = 4–6).

Because Parkin selectively translocates to the nucleus during hypoxia, we subjected HeLa cells expressing mCherry, Parkin or NLS-Parkin to hypoxia for 24 h and evaluated transcriptional changes by RNA-seq. Multi-Dimensional Scaling (MDS) revealed a marked difference in the transcriptomes of normoxic and hypoxic samples used in our study (Fig. 5a). However, in contrast to the clustering of Parkin and mCherry observed at normoxia, the transcriptome of Parkin-expressing cells shifted towards NLS-Parkin during hypoxia indicating a dose-wise effect for nuclear Parkin on hypoxic gene expression (Fig. 5a). Along these lines, despite no role for Parkin on basal gene expression (see Fig. 4c,d), Parkin altered the expression of 158 genes (fold-change ≥ 1.5, FDR < 0.05) during hypoxia, of which 58 (37%) were shared with the NLS-Parkin transcriptome (Fig. 5b). Consistent with a dose-dependent effect, NLS-Parkin significantly altered the abundance of 585 transcripts during hypoxia (Fig. 5b). To exclude indirect transcriptional changes and identify the potential direct downstream effects of Parkin in the nucleus, we focused our attention on these overlapping DEGs shared by Parkin and NLS-Parkin during hypoxia. Importantly, all 58 DEGs within this subset were up (33%) or downregulated (67%) similarly in Parkin and NLS-Parkin cells highlighting the specificity of this pool of Parkin-dependent transcripts (Fig. 5b). Hierarchal clustering of these shared hypoxia-responsive DEGs demonstrated similar magnitudes of change among Parkin and NLS-Parkin replicates (Fig. 5c). Pathway analysis revealed significant enrichment of HIF-1 signaling, the heat shock protein response, and various metabolic processes (Fig. 5d). Using the chromatin immunoprecipitation enrichment (ChEA) tool within EnrichR, we examined potential upstream regulators of the overlapping DEG subset shared by Parkin and NLS-Parkin. Similar to our analysis of NLS-mediated DEGs during baseline normoxic conditions (see Suppl Fig. S4), several nuclear receptors known to regulate metabolic gene expression emerged as potential transcriptional regulators of the shared hypoxia-responsive DEGs among Parkin and NLS-Parkin (Fig. 5e). Finally, we also confirmed the increased and decreased transcript levels of select genes identified in the RNA-seq by qPCR analysis (Fig. 5f). Collectively, these data demonstrate that Parkin can function both as a positive and negative regulator of gene expression during hypoxia and suggest that downstream induction of metabolic transcripts may occur through interaction with one or more nuclear receptors.

### Nuclear Parkin interacts with ERRα and enhances its transcriptional activity

**Figure 6:**
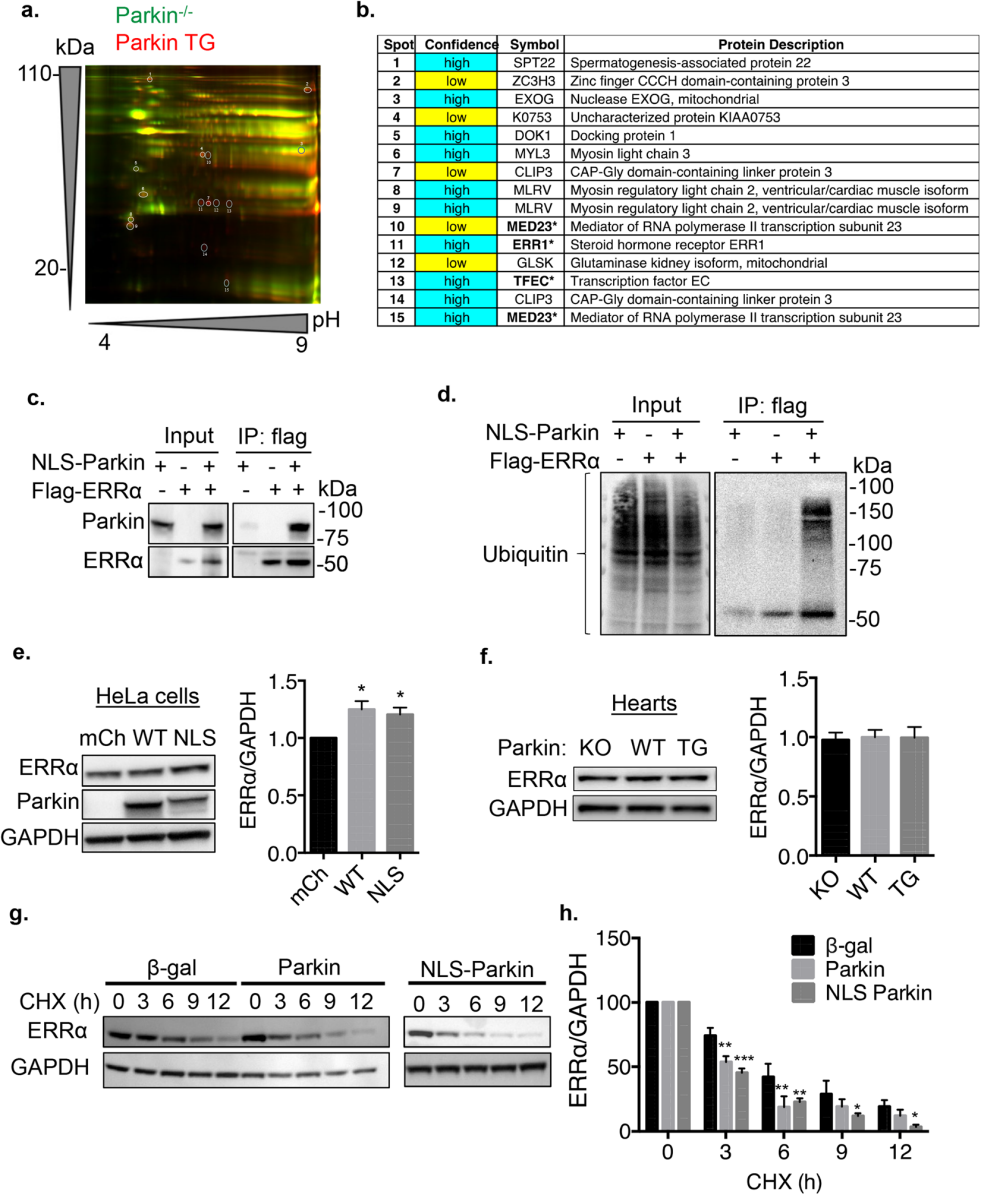
Nuclear Parkin interacts with ERRα. (**a**) 2-D gel image of proteins pulled down from Parkin^−/−^ vs Parkin TG heart lysates using agarose conjugated TUBES. Circles highlight spots picked for identification. (**b**) Protein spots identified by LC-MS/MS. Text in bold plus asterisk indicate a nuclear protein. (**c**) HeLa cells were transfected with NLS-Parkin and Flag-ERRα as indicated. Western blot analysis of input and anti-flag immunoprecipitates for ERRα and NLS-Parkin. (**d**) Representative Western blot of input and anti-Flag immunoprecipitation using anti-ubiquitin. All immunoprecipitation experiments were repeated at least four different times with similar results. (**e**) Representative Western blot and quantification of protein levels of endogenous ERRα normalized to GAPDH in HeLa cells expressing mCherry, Parkin, or NLS-Parkin (n = 5, *p < 0.05 compared to mCherry (mCh)). (**f**) Representative Western blot and quantification of ERRα protein levels normalized to GAPDH in hearts from Parkin KO, WT, or Parkin TG mice (n = 3). (**g**) Representative Western blot for endogenous ERRα in HeLa cells expressing β-gal, Parkin, or NLS-Parkin after treatment with 100 ng/μl cycloheximide (CHX) for indicated time points. GAPDH was used as a loading control. (**h**) Quantification of ERRα protein levels normalized to GAPDH in HeLa cells expressing β-gal, Parkin, or NLS-Parkin and subjected to cycloheximide for indicated time points (n = 3, *p < 0.05, **p < 0.01, ***p < 0.001 vs β-gal at the same timepoint). Full length blots are available in Supplementary Figure S6.

To further assess the function of Parkin in the nucleus, we conducted a screen for novel Parkin targets in the heart. We utilized cardiac-specific Parkin transgenic (TG) mice which have increased levels of ubiquitinated proteins at baseline due to elevated Parkin^26^. We confirmed that these mice had increased levels of Parkin in the nucleus and increased ubiquitination of nuclear proteins (Suppl Fig. S5a,b). Next, we used tandem ubiquitination binding proteins (TUBES) conjugated to agarose beads to capture ubiquitinated proteins in heart lysates prepared from Parkin^−/−^ and Parkin TG mice. The Parkin^−/−^ mice were used as a negative control to select for ubiquitinated proteins unique to the Parkin TG mice with the assumption that these proteins represented potential Parkin substrates. The captured proteins were separated by 2D-gel electrophoresis and unique spots in Parkin TG samples were identified by liquid chromatography-tandem mass spectrometry (LC-MS/MS) (Fig. 6a,b). Out of the 15 spots selected for further analysis, four of these were identified to be nuclear proteins, including estrogen-related receptor alpha (ERRα). This transcription factor is involved in regulating genes involved in mitochondrial biogenesis and metabolism^27–29^.

To confirm that Parkin interacts with ERRα, we performed co-immunoprecipitation experiments in cells transfected with mCherry or NLS-Parkin plus Flag vector or Flag-ERRα. We found that NLS-Parkin co-immunoprecipitated with Flag-ERRα but not Flag alone, confirming that the two proteins exist in the same complex (Fig. 6c). To determine if Parkin ubiquitinates ERRα, we also analyzed the Flag-ERRα immunoprecipitate for presence of ubiquitinated proteins. The anti-ubiquitin detected a band above the 50 kDa which corresponds to the size of flag-tagged ERRα in the IP lanes, suggesting that overexpression of NLS-Parkin led to increased ubiquitination of ERRα (Fig. 6d). We also detected higher ubiquitinated bands which could potentially correspond to other ubiquitinated proteins in complex with ERRα-Parkin. Taken together, these data suggest that ERRα is a substrate for Parkin in the nucleus.

Ubiquitination is a very versatile process that can affect a protein’s stability, subcellular localization or activity^30^. Depending on the protein, Parkin-mediated ubiquitination can promote proteasomal degradation or stabilization of the substrate. To determine whether Parkin promotes degradation of ERRα, we performed experiments to assess the effects of Parkin on the steady-state levels and turnover of ERRα. We found that overexpression of Parkin or NLS-Parkin led to a modest, but significant, increase in ERRα protein levels in HeLa cells (Fig. 6e). However, ERRα protein levels were unaltered in hearts from Parkin-deficient mice or cardiac-specific Parkin TG mice when compared to WT hearts (Fig. 6f). Next, we investigated the effect of Parkin and NLS-Parkin on the turnover of endogenous ERRα by performing a cycloheximide (CHX) chase assay. HeLa cells overexpressing β-gal, Parkin or NLS-Parkin were treated with CHX to inhibit new protein synthesis. ERRα levels were assessed by Western blotting at different time points following addition of CHX. Unexpectedly, we found that although both Parkin and NLS-Parkin overexpression increased baseline levels of ERRα in HeLa cells, they also increased the rate of ERRα degradation (Fig. 6g,h). The half-life of ERRα decreased from ~5 h (β-gal) to ~3 h in the presence of Parkin or NLS-Parkin. Overall, these findings suggest that Parkin might increase turnover rates of ERRα.

**Figure 7:**
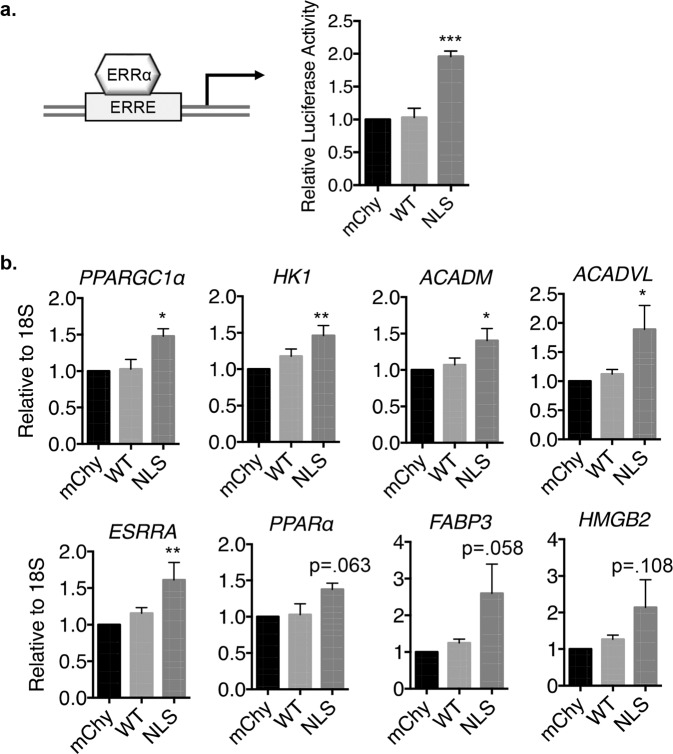
Nuclear Parkin activates ERRα-regulated gene transcription. (**a**) Quantification of relative Luciferase activity in HeLa cells transfected with mCherry, Parkin, or NLS-Parkin plus ERE/ERRE-Luciferase reporter (n = 3). (**a**) Real-time quantitative PCR of *Peroxisome Proliferator-Activated Receptor Gamma Coactivator 1-alpha* (*PPARGC1α*), *Hexokinase 1* (*HK1*), *Acyl-CoA Dehydrogenase Medium chain* (*ACADM*), *Acyl-CoA Dehydrogenase Very Long chain* (*ACADVL*), *Estrogen Related Receptor alpha* (*ESRRA*), *Peroxisome Proliferator-Activated Receptor alpha* (*PPARα*), *Fatty Acid Binding Protein 3* (*FABP3*), and *High Mobility Group Box 2* (*HMGB2*) in HeLa cells expressing mCherry, Parkin, or NLS-Parkin (n = 3–6). *p < 0.05, ***p < 0.001 compared to mCherry.

ERRα functions as a transcription factor that binds to estrogen-related receptor response elements (ERREs) in the promoter regions of target genes to induce their expression^31,32^. To determine whether nuclear Parkin affects the transcriptional activity of ERRα, we performed a luciferase reporter assay using a reporter construct containing an ERRE. We found that overexpression of NLS-Parkin, but not mCherry or Parkin, led to increased luciferase activity in HeLa cells (Fig. 7a). To further confirm the increased ERRα transcriptional activity in the presence of nuclear Parkin, we assessed the mRNA levels of several known ERRα target genes in HeLa cells. We found a significant increase in transcript levels of Peroxisome Proliferator-Activated Receptor Gamma Coactivator 1-alpha (*PPARGC1α*), Hexokinase I (*HK1*), Acyl-CoA Dehydrogenase Medium Chain (*ACADM*), and Acyl-CoA Dehydrogenase Very Long Chain (*ACADVL*) in HeLa cells overexpressing NLS-Parkin (Fig. 7b). ERRα also regulates its own transcription and we confirmed that ERRα transcript (*ESRRA*) levels were increased in cells overexpressing NLS-Parkin. Peroxisome Proliferator-Activated Receptor alpha (*PPARα*), Fatty Acid Binding Protein 3 (*FABP3*), and High Mobility Group Box 2 (*HMGB2*) transcript levels were also increased by NLS-Parkin but did not reach statistical significance. Taken together, these results suggest that nuclear Parkin also increases the transcriptional activity of endogenous ERRα.

## Discussion

The findings in this study provide important new insights into Parkin’s function in cells. Parkin is well-recognized for its role in promoting mitophagy of impaired mitochondria. In this study, we demonstrate that Parkin also has functions in the nucleus where it regulates gene transcription. Specifically, we uncovered that Parkin regulates gene transcription during oxygen limiting conditions, where hypoxia results in translocation of Parkin to the nucleus. We also discovered that nuclear-targeted Parkin causes widespread changes in gene transcription, including genes involved in mitochondrial biogenesis and various metabolic pathways. Similar changes in gene transcription are observed in cells expressing wild type Parkin after exposure to hypoxia confirming that these changes are specifically due to Parkin’s functions in the nucleus. We also found that nuclear Parkin interacts with ERRα and causes increased transcription of ERRα-target genes. Overall, our study establishes that Parkin has diverse roles in cells and suggests that the underlying pathology observed in Parkin-deficiency is not likely due solely to impaired mitophagy.

Although other studies have reported the presence of Parkin in the nucleus^33,34^, our study is the first to report that Parkin is recruited to the nucleus in response to hypoxia. It has previously been reported that Parkin can suppress gene transcription by promoting degradation of transcriptional regulators in the cytosol^11,35^. It has also been reported that Parkin exhibits DNA binding activity to suppress gene transcription, a function that is independent of its E3 ubiquitin ligase activity^36^. Here, we report that nuclear Parkin can both repress and activate gene transcription in the nucleus. Although it is possible that some of the changes in gene transcription observed by nuclear Parkin is due to direct binding of DNA, our data supports a role for Parkin-mediated ubiquitination in regulating gene transcription. First, we found that nuclear localization of Parkin leads to increased ubiquitination of nuclear proteins. The increase in the level of protein ubiquitination when Parkin is in the nucleus suggests that its E3 ubiquitin ligase activity is enhanced. Also, dysfunctional Parkin mutants are selectively excluded from the nucleus. Finally, we observed that nuclear Parkin interacts with the transcription factor ERRα, increases its transcriptional activity, and enhances transcription of its target genes. Thus, it is clear that Parkin can regulate gene transcription via multiple mechanisms, including ubiquitinating nuclear proteins involved in gene transcription as well as directly binding to DNA.

Our findings in this study suggest that Parkin has several potential substrates in the nucleus that have yet to be identified and validated. Although our transcriptomic analyses centered around upstream transcription factors and coactivators, other proteins in the nucleus, such as histones, also affect gene transcription. Histones are subjected to various post-translational modifications that influence gene expression^37^. Histone ubiquitination, for example, has been linked to both repression and activation of transcription^38–40^. Whether histones are Parkin substrates, and what effect it has on the transcriptome is an interesting avenue of research that should be addressed in future studies.

Moreover, our data suggest that wild type Parkin is not active in the nucleus at baseline in HeLa cells. Although some of Parkin localizes to the nucleus when overexpressed, the RNA seq results and qPCR analysis for specific ERRα targets clearly show that Parkin has little effect on gene transcription at baseline. This is consistent with previous studies on Parkin-mediated mitophagy where overexpression of Parkin does not induce autophagy of mitochondria unless an additional stress is added, such as treatment with a mitochondrial uncoupler^15,41^. However, we identified that Parkin translocates to the nucleus during hypoxia which correlates with transcriptional changes in a significant number of genes. Because many different pathways involved in regulating transcription are activated by hypoxia, it was unclear which changes were specifically due to Parkin’s action in the nucleus. Therefore, utilizing a nuclear targeted Parkin construct to restrict its subcellular localization to the nucleus allowed us to identify the genes that were changed by wild type Parkin in the nucleus during hypoxia. Although we found that NLS-Parkin had some effect on gene activation at baseline, the changes in these genes were further amplified by hypoxia, suggesting that the oxygen deprivation contributes to enhanced activation of NLS-Parkin. Specifically, we identified changes in 58 genes that were common to both wild type Parkin and NLS-Parkin. Thus, comparing wild type Parkin plus hypoxia to NLS-Parkin allowed us to identify the genes that were specifically changed by nuclear Parkin. The RNA sequencing also identified changes in 90 genes that were unique to cells overexpressing wild type Parkin during hypoxia and were not changed by NLS-Parkin. Parkin also is present in the cytosol during hypoxia and the changes in those genes are most likely due to Parkin’s action (directly or indirectly) on transcriptional regulators that normally reside in the cytosol.

Despite having different effects on gene transcription at baseline, we found that both wild type and NLS-Parkin overexpression led to a modest but significant increase in ERRα protein levels while simultaneously increasing the rate of ERRα degradation in HeLa cells. This suggests that Parkin might be increasing the turnover of ERRα in cells. Ren *et al*. previously reported that Parkin interacts with ERRα and promotes the degradation of ERRα, β and γ^35^. This group also found that ERRα stability was increased in the absence of Parkin and that this correlated with increased levels of its target genes *Monoamine Oxidase A* and *B* in brain sections^35^. Although we observed similar effects on ERRα stability, we observed the opposite effect on gene transcription. Currently, the reason for the different findings between the two studies are currently unclear but is likely due to differences in experimental models. Both studies used HeLa cells to examine the effect of Parkin on ERRα degradation and similarly found that Parkin increased the rate of degradation. However, in studies assessing the effect of Parkin on gene expression, we performed our experiments in HeLa cells while Ren *et al*. assessed changes in gene expression in SH-SY5Y cells and midbrain neuronal cultures^35^. Also, both studies used the same Parkin-deficient mice^42^ but assessed ERRα levels in different tissues (brain vs heart). The fact that Parkin increased ERRα protein levels in HeLa cells but not heart tissue in our studies suggests tissue-specific differences. Thus, additional studies are needed to evaluate the relationship between Parkin and ERRα, and to determine the cell and tissue-specific effects of nuclear Parkin.

We also observed that Parkin was sometimes detected as a double band in our Western blotting experiments depending on the condition. For instance, we found that Parkin was detected as a double band in HeLa cells with stable overexpression of YFP-Parkin, while transient overexpression of Parkin resulted in a single band. We also noted a similar Parkin band pattern when analyzing Parkin in various tissues. Interestingly, the heavier Parkin band seemed to be selectively increased in the nuclear fractions. It is likely that the higher molecular weight band represents post translationally modified Parkin. It has previously been reported that Parkin is autoubiquitinated upon activation^43–45^. Parkin is also phosphorylated by PINK1 on Ser65 to activate mitophagy^20,46,47^. Currently, our knowledge of Parkin’s posttranslational modifications are still limited and whether autoubiquitination and/or phosphorylation are involved in activating the nuclear translocation of Parkin clearly requires further investigation.

Finally, there is little evidence that loss of mitophagy is solely responsible for the mitochondrial dysfunction in dopaminergic neurons in PD and in cardiac tissue after a myocardial infarction. Instead, it is likely that loss of Parkin’s nuclear functions also contributes to the underlying pathology observed in PD and after myocardial injury. The fact that Parkin mutants identified in PD are excluded from the nucleus suggests that its nuclear function is also disrupted in PD patients. In support of this notion, a recent analysis of the transcriptomes in fibroblasts from PD patients showed that Parkin gene mutations were associated with deregulation of many genes, including those involved in metabolic pathways^48^. Interestingly, a study on transcriptomic alterations in skin biopsies from patients with Parkinson’s Disease also noted changes in various metabolic pathways, nuclear function, tumorigenesis, and immune regulation^49^. A similar study analyzing the transcriptomes in fibroblasts from PD patients with Parkin gene mutations revealed altered metabolic pathways, specifically amino acid and folate^48^. Our findings confirm that Parkin’s role in the pathogenesis of PD is more complex than its role in mitophagy.

Many studies have demonstrated that Parkin plays a key role in maintaining mitochondrial function and cellular homeostasis which has primarily been attributed to its role in regulating mitophagy. However, Parkin’s functions are clearly more complex and diverse than initially thought. Parkin’s functions appear to depend on stressors present in the cell, and future studies need to focus on physiological stressors in cells and *in vivo*. Parkin is considered an attractive therapeutic target to improve mitochondrial health, especially in PD patients. However, a deeper understanding of the diverse functions of Parkin is critical before we can consider it a viable therapeutic target.

## Methods

### Mouse models

All experimental procedures were performed in accordance with institutional guidelines and approved by the Institutional Animal Care and Use Committee of the University of California, San Diego. Parkin KO mice were obtained from Jackson Laboratories (B6. 129S4-Park2tm1Shn/J, stock #006582) and their cardiac phenotype has been characterized previously^5^. Parkin transgenic mice (C57Bl/6 background) overexpress human Parkin under the α-myosin heavy chain (α-MHC) promoter^26^. Both male and female mice (8–12 weeks) were used for these experiments.

Myocardial infarctions were carried out on 8–10 week old C57/B6J male mice by permanently ligating the left anterior descending coronary artery as described previously^5^. Mice were anesthetized with isoflurane, intubated, and ventilated. An 8–0 silk suture was placed around the left anterior descending coronary artery and then tightened. Infarction was confirmed by blanching of anterior left myocardium wall. The suture was left in place, and the animal was immediately closed up.

### Cell culture

SV40 transformed mouse embryonic fibroblasts (MEFs) and HeLa cells were maintained in Dulbecco’s modified Eagle’s medium (DMEM, ThermoFisher) containing 10% fetal bovine serum (FBS, ThermoFisher) and antibiotics (100 U/mL penicillin and 100 µg/mL streptomycin). Neonatal rat ventricular myocytes (NRVMs) were prepared via enzymatic digestion of hearts from 1- to 2-day-old Sprague-Dawley rats and plated in DMEM and M199 in a 4:1 ratio (Life Technologies) plus 10% FBS and antibiotics. HeLa cells stably expressing YFP-Parkin and PINK1^−/−^ HeLa cells have been described previously^15,50^ and were generously provided by Dr. Richard Youle. For hypoxia experiments, cells were cultured in media supplemented with 20 mM HEPES pH 7.4 (ThermoFisher) and placed into BD GasPak EZ pouches (BD Biosciences). Nutrient deprivation was carried out by incubating cells in DMEM lacking glucose and serum.

### Plasmids and adenoviral constructs

The pCMV Flag ERRα was a gift from Toren Finkel (Addgene plasmid #10975)^51^. The mCherry-Parkin, mCherry-ParkinR42P and mCherry-ParkinG430D constructs and adenoviruses have been previously described^5^. NLS-Parkin was generated by linking mCherry and three consecutive SV40 nuclear localization signals (pmCherry-NLS was a gift from Martin Offterdinger^52^, Addgene plasmid #39319) to the N-terminal end of Parkin. Cells were transiently transfected with DNA using Fugene 6 Transfection Reagent (Promega) according to the manufacturer’s instructions. Cells were infected with adenoviruses in DMEM + 2% heat-inactivated serum and rescued with their respective culture media. All experiments were performed 24 h after infection or transfection.

### Subcellular fractionation

Tissues were homogenized in ice cold isolation buffer (250 mM sucrose, 5 mM KH_2_PO_4_, 2 mM MgCl_2_, 10 mM MOPS, 1 mM EGTA, 0.1% fatty-acid free BSA, pH7.4) and Complete protease inhibitor (Roche Applied Bioscience) and cleared of debris by centrifugation at 200 × g for 10 min. Supernatant was subjected to centrifugation at 800 × g for 15 min to separate the cytosol and the nuclei. The cytosol was further cleared by centrifugation at 20,000 × g for 15 min, while the nuclear pellet nuclei was resuspended in isolation buffer and recovered by centrifugation at 800 × g for 15 min. The nuclear pellet was solubilized in RIPA buffer containing 50 mM Tris-HCl pH 8.0, 150 mM NaCl, 1% Triton X-100, 0.5% sodium deoxycholate, 0.1% SDS, 1 mM EGTA, 1 mM EDTA, and Complete protease inhibitor (Roche Applied Bioscience). The nuclear isolation from HeLa cells was adapted from a previously published protocol^53^. HeLa cells were disrupted by sonication in ice cold buffer C (10 mM HEPES pH 7.6, 10 mM NaCl, 1.5 mM MgCl_2_, 10% glycerol, 0.2% NP40). The cell homogenate was subjected to centrifugation at 22,000 × g for 5 min to separate the cytosol from organelles. The pellet was resuspended in buffer containing 50 mM Tris-HCl, pH 7.4, 150 mM NaCl, 1 mM EGTA, 1 mM EDTA, 1% Triton X-100, agitated on ice for 15 min, and centrifuged for 5 min at 2,800 × g. The resulting nuclear pellet was solubilized in RIPA buffer.

### Immunoprecipitation and Western blot analysis

For immunoprecipitation, whole cell lysates were pre-cleared with protein A agarose beads for 30 min at 4 °C, and then incubated with anti-Flag (Sigma, F7425) at 4 °C for 18 h. The immunoprecipitate was captured by incubation with protein A agarose beads for 120 min. The beads were washed with ice-cold PBS and proteins eluted with SDS loading buffer by boiling for 5 min at 95 °C. For Western blot analysis, the proteins were separated on NuPAGE Bis-Tris gels (Invitrogen) and transferred to nitrocellulose membranes (GE Healthcare Life Sciences). Membranes were immunoblotted with antibodies towards: Parkin (Cell Signaling Technology), ERRα (Abcam), Flag (Sigma), Histone H3 (Cell Signaling Technology), GAPDH (GeneTex), Ubiquitin (Santa Cruz Biotechnology) and imaged using a ChemiDoc XRS + System (Bio-Rad).

### Fluorescence microscopy

Cells were fixed, permeabilized, and blocked as described previously^41^. The cells were stained with anti-TOMM20 (Sant Cruz Biotechnology, 11415) to label mitochondria and incubated with Alexa Fluor 488 or 594 secondary antibodies (Life Technologies A11037) and Hoechst 33342 (Life Technologies, H3570) to stain nuclei. Cells were imaged with a Carl Zeiss AxioObserver Z1 fitted with a motorized Z-stage. Z-stacks were acquired in ApoTome mode using a high-resolution AxioCam MRm digital camera, a 63X Oil-immersion objective and Zeiss AxioVision 4.8 software (Carl Zeiss).

### RNA-sequencing and Bioinformatics analysis

RNA-sequencing and bioinformatics analyses were conducted at the IGM Genomics Center, and the UCSD Center for Computational Biology and Bioinformatics at the University of California, San Diego, La Jolla, CA, respectively. RNA-sequencing was conducted via Illumina HiSeq. 2000. Quality control of the raw fastq files was performed using the software took FastQC v0.11.3. Sequencing reads were trimmed with Trimmomatic v0.36 and aligned to the human genome (GRCh37.p13) using the STAR aligner v2.5.3a^54^. Read quantification was performed with RSEM v 1.3.0^55^ and the Gencode release 19^56^. The R BioConductor packages edgeR and limma were used to implement the limma-voom method for differential expression analysis^57^. In brief, lowly expressed genes (counts per million (cpm) ≥ 1 in at least 4 of the samples), and then trimmed mean of M-values (TMM) normalization was applied. The values weighted for inter-subject correlations in repeated measures of samples, after which ImFit was used to fit per-gene linear models and empirical Bayes moderation was applied with the eBayes function. Significance was defined by using an adjusted p-value cut-off of 0.05 after multiple testing correction^58^ using a moderated t-statistic in limma and a minimum absolute log-fold-change threshold of 1.5.

### Quantitative PCR

RNA extraction, cDNA synthesis, and quantitative PCR were performed as described previously^59^. Briefly, RNA was isolated from cells using the RNeasy Mini Kit (Qiagen) following the manufacturers protocols. cDNA was synthesized using the QuantiTecht Reverse Transcription kit (Qiagen). Primers for *Asns*, *Hkdc1*, *Dynlt3*, *Mylk3*, *Esrra*, *Ppara*, *Acadm*, *Acadvl*, *Fabp3*, *Hk1*, *Hmgb2*, *Ppargc1a*, and *Rn18s*, and TaqMan Universal Master Mix II were purchased from Applied Biosystems/Life Technologies. qPCR was performed on a CFX96 Real-Time PCR Detection System (Bio-Rad). Relative mRNA was normalized to *Rn18s*, and fold change in gene expression was calculated using the 2(^−ΔΔCt^) method.

### Luciferase assay

The luciferase assay was conducted using a kit from Promega (E2920). HeLa cells were transiently transfected with mCherry, Parkin or NLS-Parkin plus an ERRE-luciferase reporter construct (a gift from Rebecca Riggins^32^, Addgene plasmid #37851) for 24 h and then transferred to 96-well plates (Costar 3904). After overnight incubation, cells were lysed and incubated with luciferin substrate for 1.5 h before assessing luciferase activity in a luminometer plate reader.

### TUBE-IP and target identification

These experiments were carried using a modified protocol^60^. Briefly, whole heart lysates were made fresh from Parkin^−/−^ and Parkin-TG mice, pre-cleared with unconjugated agarose beads, and combined with agarose beads bound to tandem ubiquitin binding entities (TUBES, LifeSensors), and pulled down via centrifugation. Proteins were subsequently eluted from the beads and sent to Applied Biomics (Hayward, CA) for separation on a 2-D gel, selection of unique spots and protein identification by liquid chromatography, tandem mass spectrometry (LC/MS-MS).

### Statistics

All experiments were repeated a minimum of three times. All statistical analyses were performed using GraphPad Prism 6 (GraphPad Software). Graphed data were represented as mean + /− SEM. Unpaired Student’s t test was used for two-group comparisons. One- or two-way ANOVA with post hoc comparisons by Tukey’s or Dunnett’s, paired, or unpaired t test was used for the comparisons between multiple groups, as appropriate.

## Supplementary information

Supplementary Information.

## Data Availability

The RNA sequencing data discussed in this publication are available in the NCBI Gene Expression Omnibus (http://www.ncbi.nlm.nih.gov/geo/) under the GEO series accession number GSE139989.

## References

[1] Sarraf SA (2013). Landscape of the PARKIN-dependent ubiquitylome in response to mitochondrial depolarization. Nature.

[2] Narendra DP (2010). PINK1 is selectively stabilized on impaired mitochondria to activate Parkin. PLoS Biol.

[3] Pickrell AM, Youle RJ (2015). The roles of PINK1, parkin, and mitochondrial fidelity in Parkinson’s disease. Neuron.

[4] Peker N, Donipadi V, Sharma M, McFarlane C, Kambadur R (2018). Loss of Parkin impairs mitochondrial function and leads to muscle atrophy. Am J Physiol Cell Physiol.

[5] Kubli, D. A. *et al*. Parkin protein deficiency exacerbates cardiac injury and reduces survival following myocardial infarction. *J Biol Chem* **288**, 915-926, M112.411363[pii]10.1074/jbc.M112.411363 (2013). 10.1074/jbc.M112.411363 PMC3543040 23152496

[6] Gong G (2015). Parkin-mediated mitophagy directs perinatal cardiac metabolic maturation in mice. Science.

[7] Veeriah S (2010). Somatic mutations of the Parkinson’s disease-associated gene PARK2 in glioblastoma and other human malignancies. Nat Genet.

[8] Kitada T (1998). Mutations in the parkin gene cause autosomal recessive juvenile parkinsonism. Nature.

[9] Ferreira M, Massano J (2017). An updated review of Parkinson’s disease genetics and clinicopathological correlations. Acta Neurol Scand.

[10] Greene, J. C. et al. Mitochondrial pathology and apoptotic muscle degeneration in Drosophila parkin mutants. Proc Natl Acad Sci USA 100, 4078-4083, doi:10.1073/pnas.07375561000737556100 [pii] (2003). 10.1073/pnas.0737556100 PMC153051 12642658

[11] Shin, J. H. *et al*. PARIS (ZNF746) Repression of PGC-1alpha Contributes to Neurodegeneration in Parkinson’s Disease. *Cell* **144**, 689-702, S0092-8674(11)00124-3 [pii] 10.1016/j.cell.2011.02.010 (2011). 10.1016/j.cell.2011.02.010 PMC3063894 21376232

[12] Kim, K. Y. *et al*. Parkin is a lipid-responsive regulator of fat uptake in mice and mutant human cells. *J Clin Invest* **121**, 3701-3712, 44736 [pii]10.1172/JCI44736 (2011). 10.1172/JCI44736 PMC3171101 21865652

[13] Johnson BN, Berger AK, Cortese GP, Lavoie MJ (2012). The ubiquitin E3 ligase parkin regulates the proapoptotic function of Bax. Proc Natl Acad Sci USA.

[14] Lee SB (2019). The AMPK-Parkin axis negatively regulates necroptosis and tumorigenesis by inhibiting the necrosome. Nat Cell Biol.

[15] Narendra, D., Tanaka, A., Suen, D. F. & Youle, R. J. Parkin is recruited selectively to impaired mitochondria and promotes their autophagy. *J Cell Biol* **183**, 795-803, jcb.200809125 [pii]10.1083/jcb.200809125 (2008). 10.1083/jcb.200809125 PMC2592826 19029340

[16] Matsuda, N. *et al*. PINK1 stabilized by mitochondrial depolarization recruits Parkin to damaged mitochondria and activates latent Parkin for mitophagy. *J Cell Biol* **189**, 211-221, jcb.200910140[pii]10.1083/jcb.200910140 (2010). 10.1083/jcb.200910140 PMC2856912 20404107

[17] Zhang J (2020). Loss of HIPK2 Protects Neurons from Mitochondrial Toxins by Regulating Parkin Protein Turnover. J Neurosci.

[18] Kim, Y. *et al*. PINK1 controls mitochondrial localization of Parkin through direct phosphorylation. *Biochem Biophys Res Commun* **377**, 975-980, S0006-291X(08)02084-6 [pii]10.1016/j.bbrc.2008.10.104 (2008). 10.1016/j.bbrc.2008.10.104 18957282

[19] Sha, D., Chin, L. S. & Li, L. Phosphorylation of parkin by Parkinson disease-linked kinase PINK1 activates parkin E3 ligase function and NF-kappaB signaling. *Hum Mol Genet* **19**, 352-363, ddp501 [pii]10.1093/hmg/ddp501 (2010). 10.1093/hmg/ddp501 PMC2796895 19880420

[20] Shiba-Fukushima K (2012). PINK1-mediated phosphorylation of the Parkin ubiquitin-like domain primes mitochondrial translocation of Parkin and regulates mitophagy. Sci Rep.

[21] Fedorowicz MA (2014). Cytosolic cleaved PINK1 represses Parkin translocation to mitochondria and mitophagy. EMBO Rep.

[22] Lim GG (2015). Cytosolic PTEN-induced Putative Kinase 1 Is Stabilized by the NF-kappaB Pathway and Promotes Non-selective Mitophagy. J Biol Chem.

[23] Gao J (2016). Cytosolic PINK1 promotes the targeting of ubiquitinated proteins to the aggresome-autophagy pathway during proteasomal stress. Autophagy.

[24] Nezich CL, Wang C, Fogel AI, Youle RJ (2015). MiT/TFE transcription factors are activated during mitophagy downstream of Parkin and Atg5. J Cell Biol.

[25] Kuleshov MV (2016). Enrichr: a comprehensive gene set enrichment analysis web server 2016 update. Nucleic Acids Res.

[26] Woodall, B. P. *et al*. Parkin does not prevent accelerated cardiac aging in mitochondrial DNA mutator mice. *JCI Insight* **5**, 10.1172/jci.insight.127713 (2019). 10.1172/jci.insight.127713 PMC6542612 30990467

[27] Schreiber SN (2004). The estrogen-related receptor alpha (ERRalpha) functions in PPARgamma coactivator 1alpha (PGC-1alpha)-induced mitochondrial biogenesis. Proc Natl Acad Sci USA.

[28] Singh, B. K. *et al*. Thyroid hormone receptor and ERRalpha coordinately regulate mitochondrial fission, mitophagy, biogenesis, and function. *Sci Signal* **11**, 10.1126/scisignal.aam5855 (2018). 10.1126/scisignal.aam5855 29945885

[29] Kim SY (2018). ESRRA (estrogen-related receptor alpha) is a key coordinator of transcriptional and post-translational activation of autophagy to promote innate host defense. Autophagy.

[30] Komander D, Rape M (2012). The ubiquitin code. Annu Rev Biochem.

[31] Zirngibl RA, Chan JS, Aubin JE (2008). Estrogen receptor-related receptor alpha (ERRalpha) regulates osteopontin expression through a non-canonical ERRalpha response element in a cell context-dependent manner. J Mol Endocrinol.

[32] Heckler MM, Thakor H, Schafer CC, Riggins RB (2014). ERK/MAPK regulates ERRgamma expression, transcriptional activity and receptor-mediated tamoxifen resistance in ER+ breast cancer. FEBS J.

[33] Cookson MR (2003). RING finger 1 mutations in Parkin produce altered localization of the protein. Hum Mol Genet.

[34] Horowitz JM, Myers J, Vernace VA, Stachowiak MK, Torres G (2001). Spatial distribution, cellular integration and stage development of Parkin protein in Xenopus brain. Brain Res Dev Brain Res.

[35] Ren Y, Jiang H, Ma D, Nakaso K, Feng J (2011). Parkin degrades estrogen-related receptors to limit the expression of monoamine oxidases. Hum Mol Genet.

[36] Sunico CR (2013). S-Nitrosylation of parkin as a novel regulator of p53-mediated neuronal cell death in sporadic Parkinson’s disease. Mol Neurodegener.

[37] Rothbart SB, Strahl BD (2014). Interpreting the language of histone and DNA modifications. Biochim Biophys Acta.

[38] Bhatnagar S (2014). TRIM37 is a new histone H2A ubiquitin ligase and breast cancer oncoprotein. Nature.

[39] Zhang Z (2017). Role of remodeling and spacing factor 1 in histone H2A ubiquitination-mediated gene silencing. Proc Natl Acad Sci USA.

[40] Lan X (2016). USP44 Is an Integral Component of N-CoR that Contributes to Gene Repression by Deubiquitinating Histone H2B. Cell Rep.

[41] Hammerling BC (2017). A Rab5 endosomal pathway mediates Parkin-dependent mitochondrial clearance. Nat Commun.

[42] Goldberg, M. S. *et al*. Parkin-deficient mice exhibit nigrostriatal deficits but not loss of dopaminergic neurons. *J Biol Chem* **278**, 43628-43635, 10.1074/jbc.M308947200M308947200 [pii] (2003). 10.1074/jbc.M308947200 12930822

[43] Trempe JF (2013). Structure of parkin reveals mechanisms for ubiquitin ligase activation. Science.

[44] Burchell L, Chaugule VK, Walden H (2012). Small, N-terminal tags activate Parkin E3 ubiquitin ligase activity by disrupting its autoinhibited conformation. PLoS One.

[45] He J (2018). PICK1 inhibits the E3 ubiquitin ligase activity of Parkin and reduces its neuronal protective effect. Proc Natl Acad Sci USA.

[46] Kondapalli C (2012). PINK1 is activated by mitochondrial membrane potential depolarization and stimulates Parkin E3 ligase activity by phosphorylating Serine 65. Open Biol..

[47] Ordureau A (2014). Quantitative proteomics reveal a feedforward mechanism for mitochondrial PARKIN translocation and ubiquitin chain synthesis. Mol Cell.

[48] Gonzalez-Casacuberta I (2018). Transcriptional alterations in skin fibroblasts from Parkinson’s disease patients with parkin mutations. Neurobiol Aging.

[49] Planken A (2017). Looking beyond the brain to improve the pathogenic understanding of Parkinson’s disease: implications of whole transcriptome profiling of Patients’ skin. BMC Neurol.

[50] Lazarou M (2015). The ubiquitin kinase PINK1 recruits autophagy receptors to induce mitophagy. Nature.

[51] Ichida M, Nemoto S, Finkel T (2002). Identification of a specific molecular repressor of the peroxisome proliferator-activated receptor gamma Coactivator-1 alpha (PGC-1alpha). J Biol Chem.

[52] Micutkova L (2012). Analysis of the cellular uptake and nuclear delivery of insulin-like growth factor binding protein-3 in human osteosarcoma cells. Int J Cancer.

[53] Miyamoto S (2010). PHLPP-1 negatively regulates Akt activity and survival in the heart. Circ Res.

[54] Dobin A (2013). STAR: ultrafast universal RNA-seq aligner. Bioinformatics.

[55] Li B, Dewey CN (2011). RSEM: accurate transcript quantification from RNA-Seq data with or without a reference genome. BMC Bioinformatics.

[56] Frankish A (2019). GENCODE reference annotation for the human and mouse genomes. Nucleic Acids Res.

[57] Law, C. W. *et al*. RNA-seq analysis is easy as 1-2-3 with limma, Glimma and edgeR. *F1000Res* **5**, 10.12688/f1000research.9005.3 (2016). 10.12688/f1000research.9005.1 PMC4937821 27441086

[58] Benjamini Y, Drai D, Elmer G, Kafkafi N, Golani I (2001). Controlling the false discovery rate in behavior genetics research. Behav Brain Res.

[59] Orogo AM (2015). Accumulation of Mitochondrial DNA Mutations Disrupts Cardiac Progenitor Cell Function and Reduces Survival. J Biol Chem.

[60] Rubel CE (2013). Diggin’ on u(biquitin): a novel method for the identification of physiological E3 ubiquitin ligase substrates. Cell Biochem Biophys.

