# Supplementary material for: Nuclear Parkin Activates the ERRα Transcriptional Program and Drives Widespread Changes in Gene Expression Following Hypoxia
Source: Sci Rep. 2020 May 22;10:8499. doi: 10.1038/s41598-020-65438-7 (PMC7244578; doi:10.1038/s41598-020-65438-7)
Supplement: Supplementary file 1 — Supplementary Information. [file 41598_2020_65438_MOESM1_ESM.pdf]

**Supplementary information for Scientific Reports**

Title: "Nuclear Parkin Activates the ERRα Transcriptional Program and Drives Widespread Changes in Gene Expression Following Hypoxia"

Sarah E. Shires[1], Justin M. Quiles[1], Rita H. Najor[1], Leonardo J. Leon[1], Melissa Q. Cortez[1], Mark A. Lampert[1], Adam Mark[2], and Åsa B. Gustafsson[1]*

[1]Skaggs School of Pharmacy and Pharmaceutical Sciences, Department of Pharmacology, Department of Medicine, University of California, San Diego, La Jolla, CA.

[2]Center for Computational Biology & Bioinformatics, Department of Medicine, University of California, San Diego, La Jolla, CA.

*Corresponding author: abgustafsson@ucsd.edu

## Supplemental Figure S1

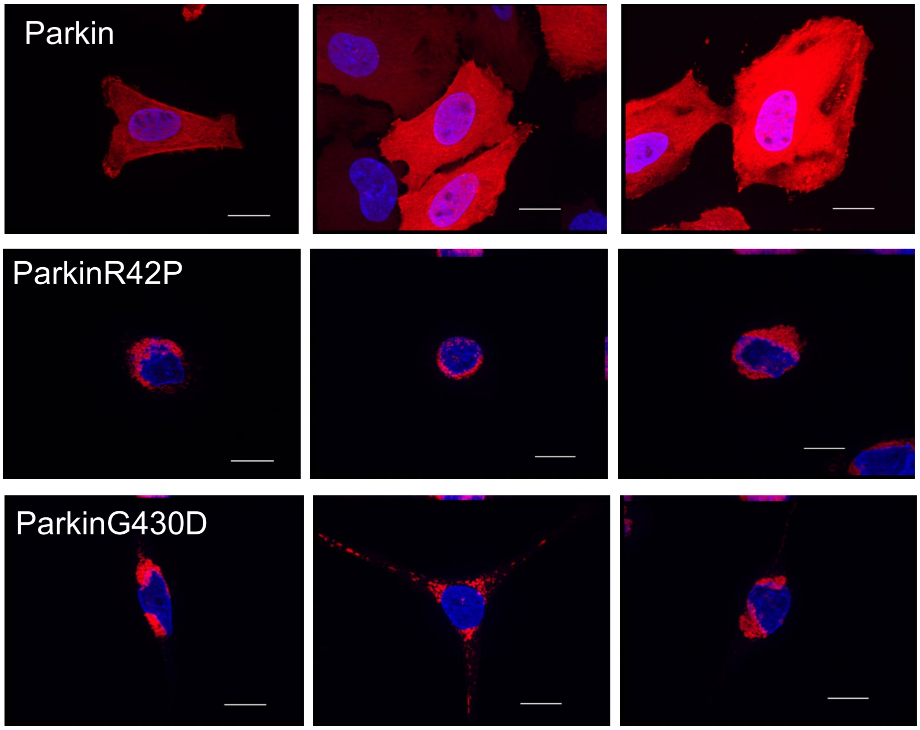


**Supplemental Figure S1.** Representative fluorescence images of HeLa cells expressing mCherry-Parkin, mCherry-ParkinR42P, or mCherry-ParkinG430D. Scale bar = 20 μm.

**Supplemental Figure S2**

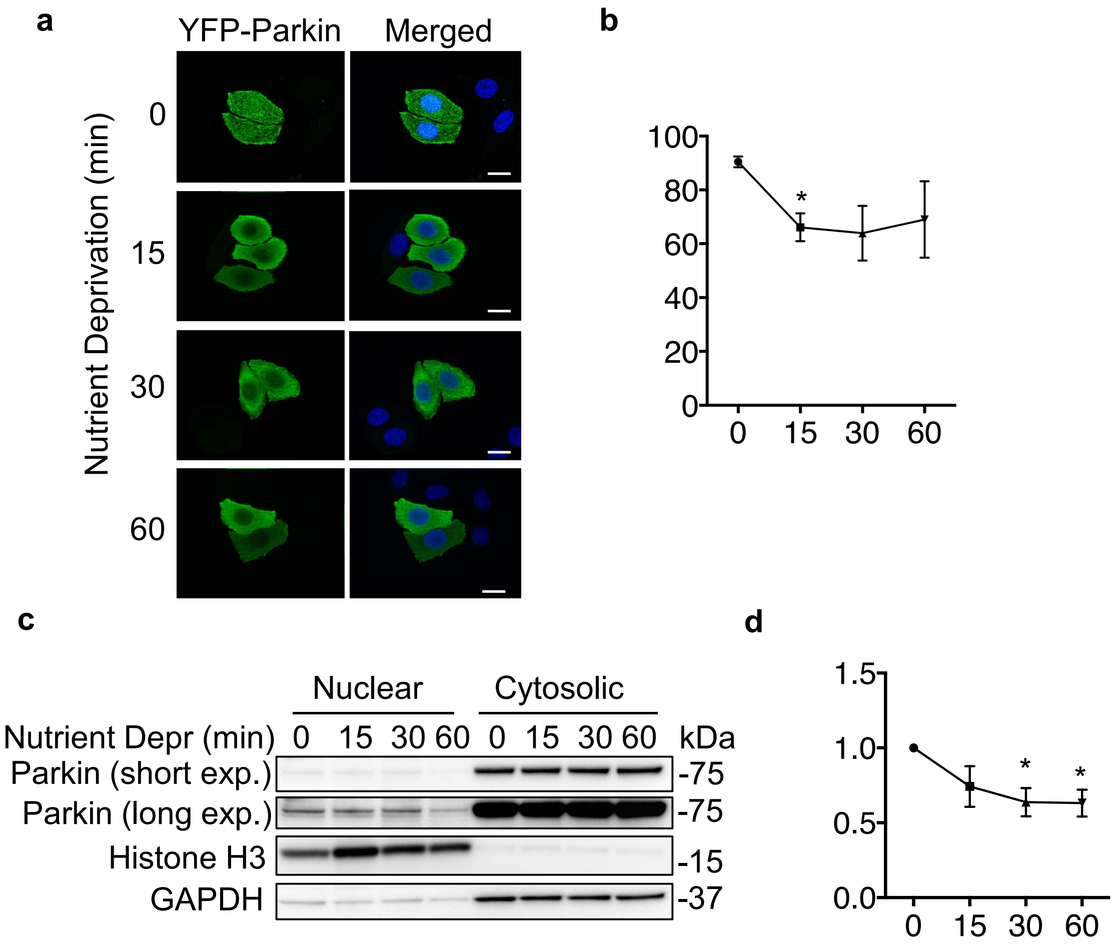


**Supplemental Figure S2.** Parkin exits the nucleus in response to nutrient deprivation. **A.** Representative images of YFP-Parkin in HeLa cells during nutrient deprivation in a time course experiment. **B.** Quantification of the number of HeLa cells with YFP-Parkin present in the nucleus. A minimum of 100 cells counted per condition in three independent experiments (n=3). **C.** Representative Western blots of nuclear and cytosolic fractions prepared from HeLa cells. **D.** Quantification of nuclear Parkin levels relative to cytosolic levels in HeLa cells subjected to nutrient deprivation (n=3). Scale bars = 20 µm, $^{*}p<0.05$ compared to 0 min.

**Supplemental Figure S3**

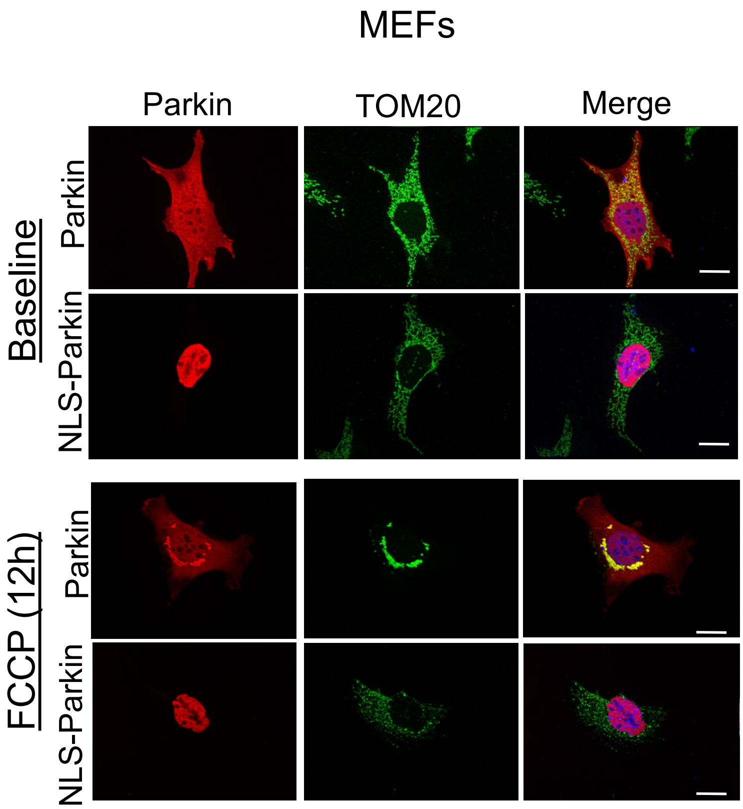


**Supplemental Figure S3.** Cellular localization of NLS-Parkin in MEFs. Representative fluorescence images of MEFs expressing Parkin or NLS-Parkin at baseline (top) and after treatment with 25 µM FCCP for 12 h (bottom). Scale bars = 20 µm.

## Supplemental Figure S4

**a**

| Regulator | P-value | Combined score |
|---|---|---|
| IRF8 | 0.00005635 | 51.64 |
| IRF1 | 0.0001667 | 33.02 |
| SOX2 | 0.0003034 | 14.29 |
| NUCKS1 | 0.0004571 | 21.8 |
| SMAD3 | 0.00237 | 12.7 |
| TP63 | 0.004579 | 48.09 |
| SETDB1 | 0.005519 | 8.58 |
| TFAP2C | 0.006005 | 9.62 |
| FOXP2 | 0.006224 | 10.51 |
| DNAJC2 | 0.008904 | 9.38 |
| TCF21 | 0.009041 | 7.56 |
| FOXA1 | 0.009041 | 7.56 |
| SCL | 0.01988 | 11.11 |
| MYB | 0.02386 | 6.75 |
| NANOG | 0.02513 | 6.79 |
| TCF7L2 | 0.02568 | 6.39 |
| **PPARG** | 0.02756 | 5.77 |
| MAF | 0.02824 | 5.31 |
| KLF6 | 0.02824 | 5.31 |
| KDM2B | 0.02824 | 5.31 |
| MYC | 0.02824 | 5.31 |
| **ESR1** | 0.04362 | 8.18 |
| ATF3 | 0.04368 | 4.43 |
| STAT3 | 0.04478 | 4.55 |
| JUN | 0.04697 | 4.37 |

**b**

| Regulator | P-value | Combined score |
|---|---|---|
| **NCOA1** | 1.51E-11 | 159.74 |
| SPDEF | 1.64E-11 | 168.96 |
| PBX3 | 4.36E-10 | 115.14 |
| **NCOA1** | 1.05E-08 | 96.75 |
| KLF9 | 1.22E-08 | 95.02 |
| STAT1 | 1.25E-08 | 117.73 |
| IRF1 | 2.60E-08 | 92.28 |
| DUX4 | 2.90E-08 | 105.41 |
| REST | 6.22E-08 | 88.28 |
| KMT2A | 1.92E-07 | 87.98 |
| ZEB1 | 5.17E-07 | 58.75 |
| KMT2A | 5.78E-07 | 81.13 |
| HSF1 | 9.95E-07 | 68.55 |
| POU1F1 | 0.00000127 | 65.97 |
| CTCF | 0.000001598 | 75.36 |
| FOSL1 | 0.000001717 | 68.03 |
| FOSL1 | 0.000001847 | 67.21 |
| IRF9 | 0.000002648 | 76.44 |
| POU5F1 | 0.00000557 | 74.62 |
| HSF1 | 0.000007417 | 63.13 |
| SOX2 | 0.00000932 | 60.43 |
| TWIST1 | 0.00000948 | 54.89 |
| LIN28 | 0.00001072 | 87.19 |
| ZNF207 | 0.00001156 | 48.87 |
| TP63 | 0.00001378 | 62.26 |

**c**

| Regulator | P-value | Combined score |
|---|---|---|
| **PPARA** | 0.02717 | 9.5 |
| HINFP | 0.03115 | 4.74 |
| SNAI2 | 0.03122 | 5.49 |
| SNAI1 | 0.03122 | 5.49 |
| TCF3 | 0.03122 | 5.49 |
| ZNF281 | 0.03853 | 5.5 |
| SND1 | 0.04079 | 4.91 |
| TFAP2C | 0.04367 | 4.77 |

**D**

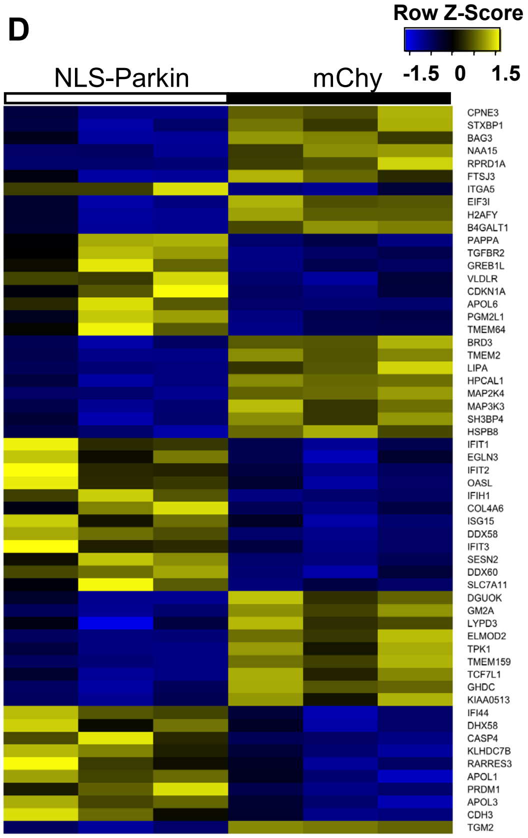


**Supplemental Figure S4.** Potential upstream transcriptional regulators of nuclear-Parkin-responsive genes. EnrichR output from: **A.** ChIP Enrichment Analysis (ChEA), **B.** TF Perturbations Followed by Expression, and **C.** TRANSFAC and JASPAR Position Weighted Matrices (PWMs) showing the top 25 most significantly enriched transcriptional regulators associated with the differentially expressed genes (DEGs) comparing HeLa cells expressing NLS-Parkin vs. mCherry. Nuclear receptors and cofactors are marked in bold, red text. **D.** Heatmap of DEGs associated with highlighted nuclear receptors and cofactors from HeLa cells expressing NLS-Parkin or mCherry.

## Supplemental Figure S5

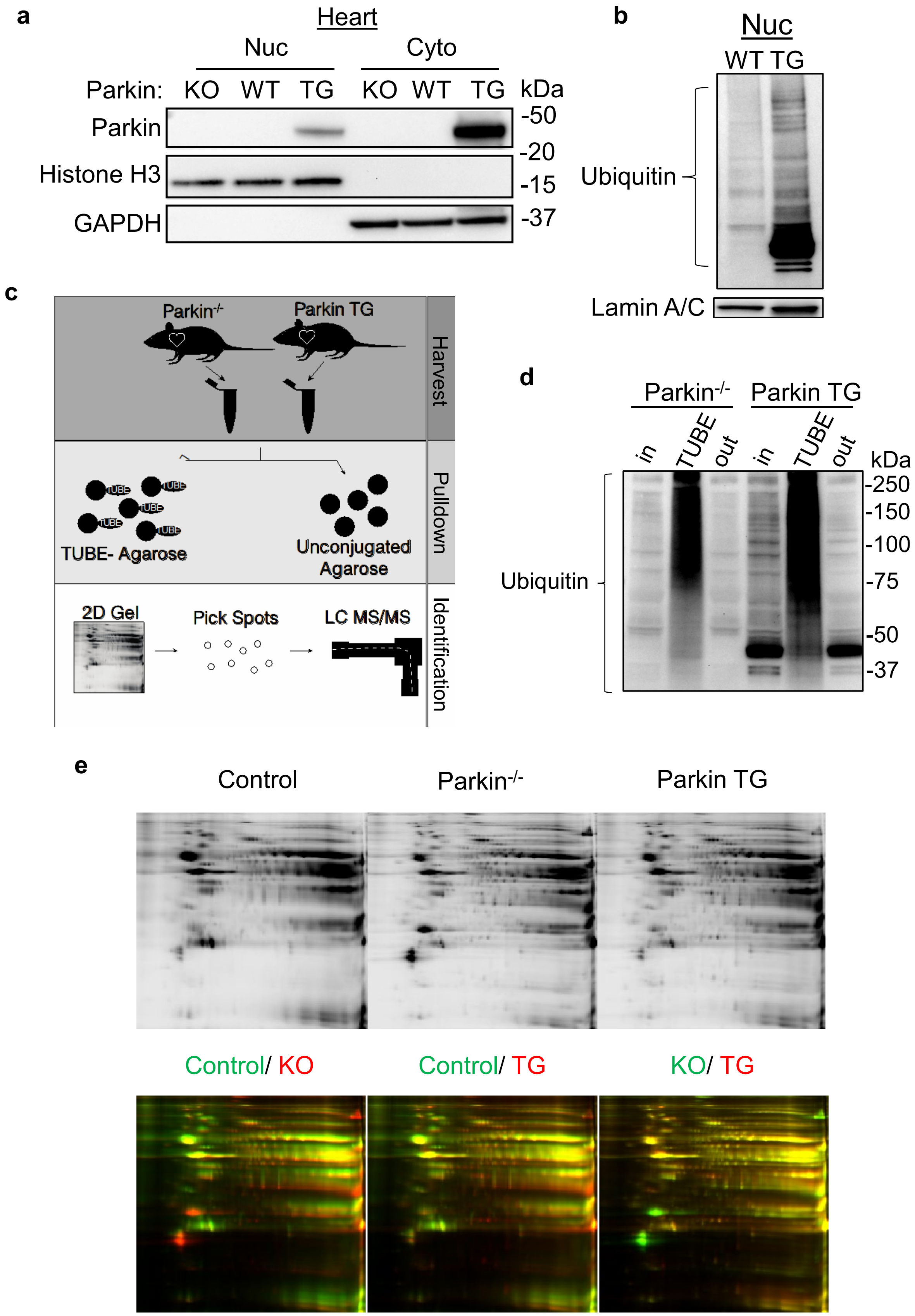

**Supplemental Figure S5.** Screen for novel Parkin targets in heart lysates **A.** Representative Western blot for Parkin in the crude nuclear and cytosolic fractions from hearts from Parkin KO, WT, and TG mice. **B.** Representative Western blot of ubiquitinated proteins in the nuclear fraction from hearts from WT and Parkin TG mice **C.** Diagram of experimental setup. Lysates were generated from the hearts of Parkin$^{-/-}$ and Parkin TG mice. Ubiquitinated proteins were pulled down using TUBE conjugated to agarose from pre-cleared lysates. The proteins were eluted from TUBE-agarose beads and separated by 2-D gel electrophoresis. Spots representing proteins pulled down in the Parkin TG sample that were not in the Parkin KO sample were identified by software analysis, proteins were identified by LC-MS/MS. **D.** Representative Western blots of heart lysates from Parkin$^{-/-}$ and Parkin TG. The input is labeled as "pre", pulldown is labeled as "TUBE", and lysate post-pulldown is labeled as "post". **E.** 2-D gel images comparing TUBE pulldowns from agarose control, Parkin KO, and Parkin TG.

## Supplemental Figure S6 – Full length blots

### Figure 1G

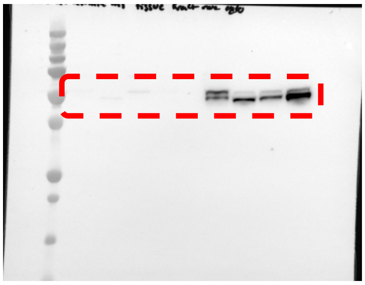

Parkin (short)

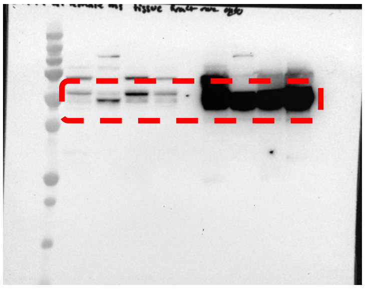

Parkin (long)

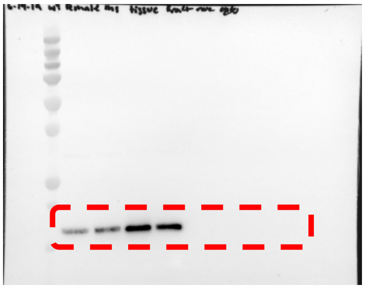

Histone H3

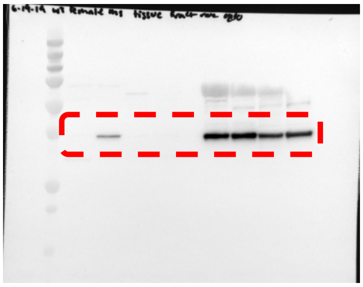

GAPDH

### Figure 1H

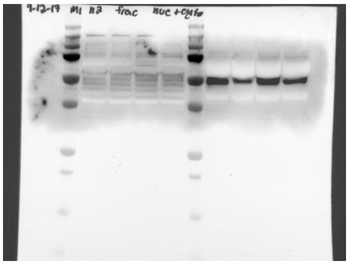

Parkin

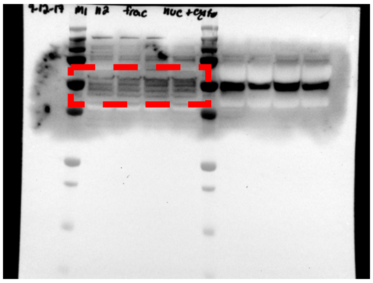

Parkin (darker)

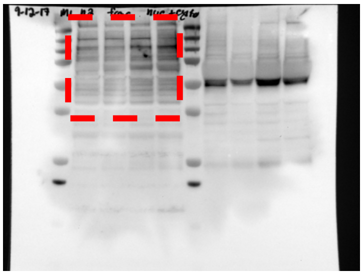

Ubiquitin

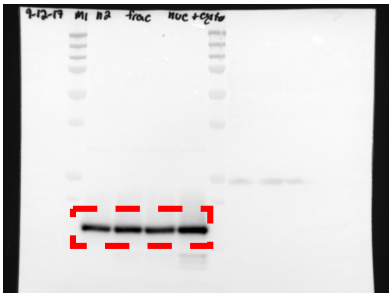

Histone H3

**Figure 2C**

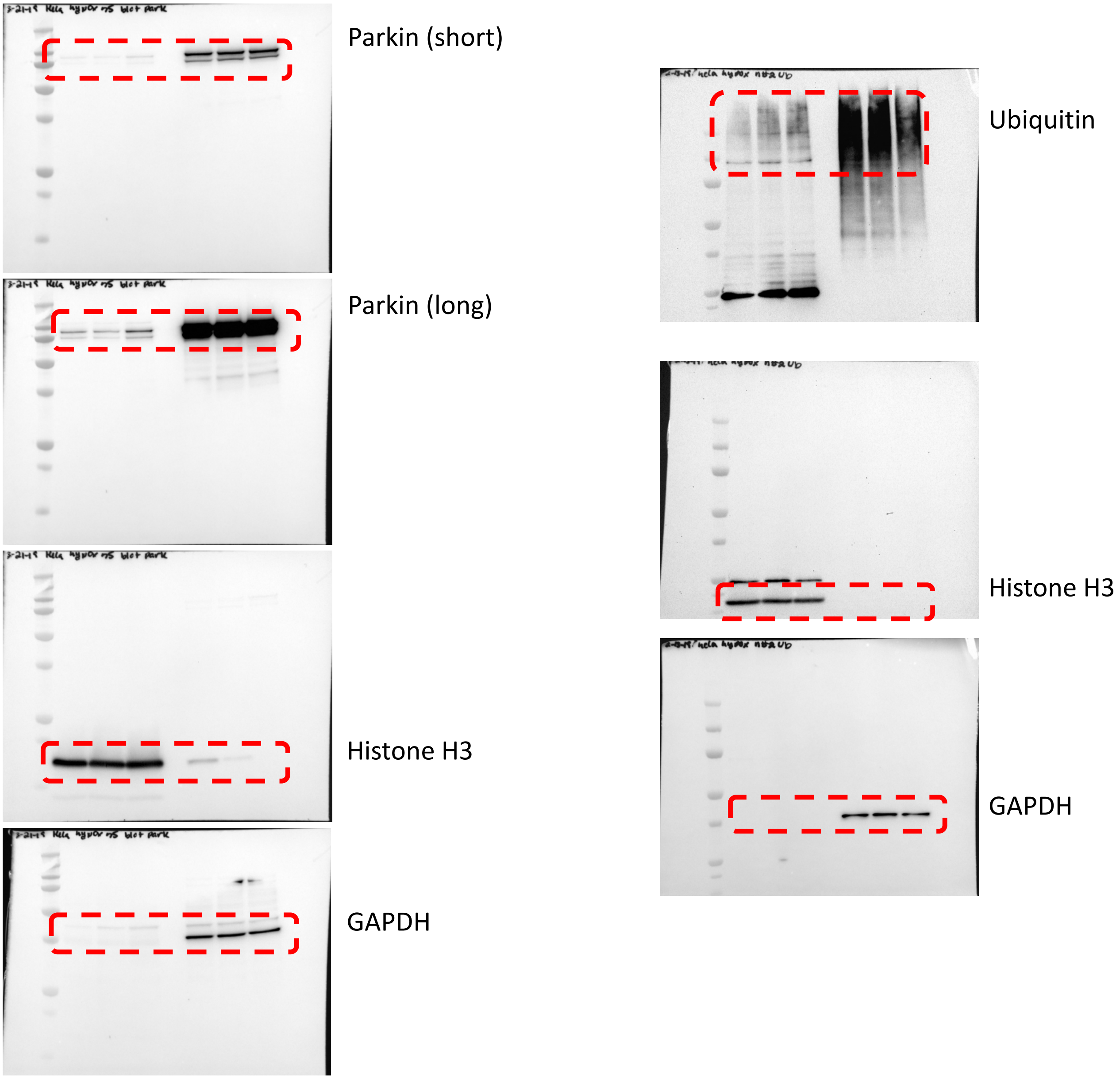
Parkin (short)
Parkin (long)
Histone H3
GAPDH
Ubiquitin
Histone H3
GAPDH

**Figure 6C**

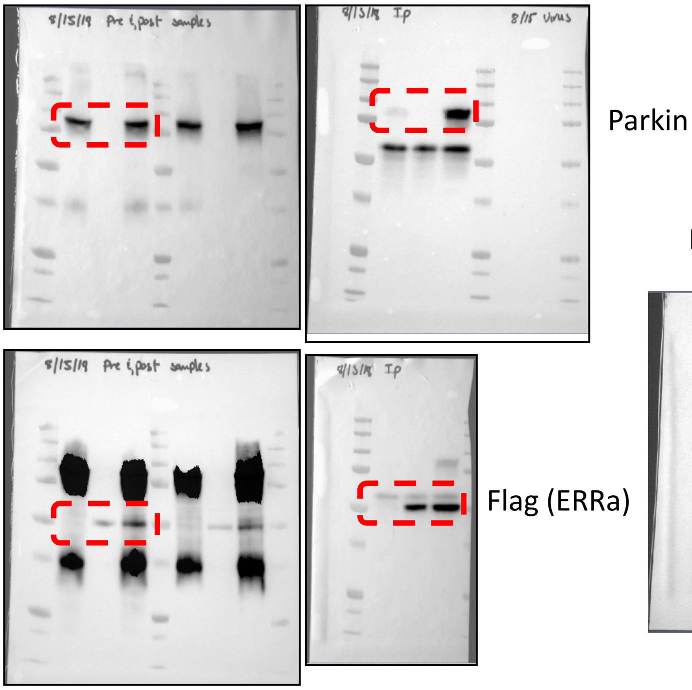


**Figure 6D**

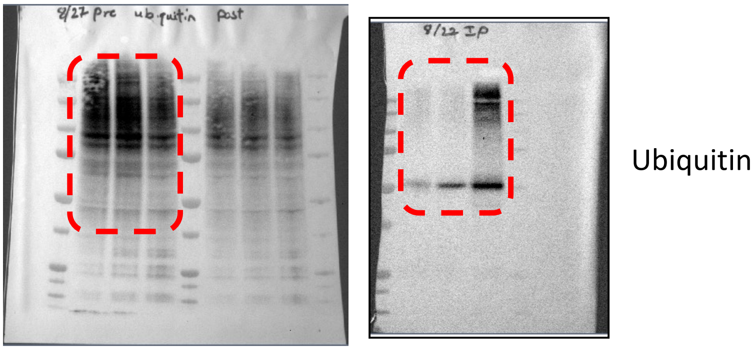


**Figure 6E**

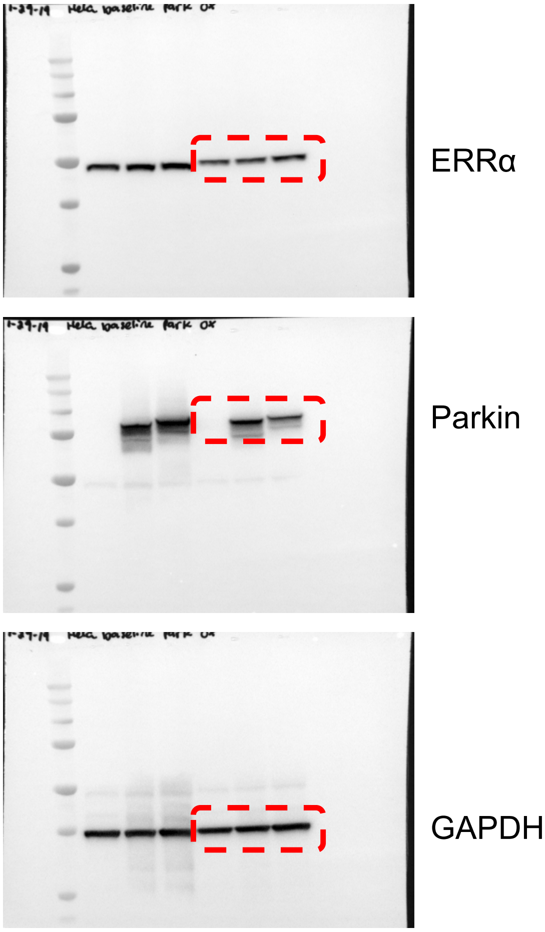


**Figure 6F**

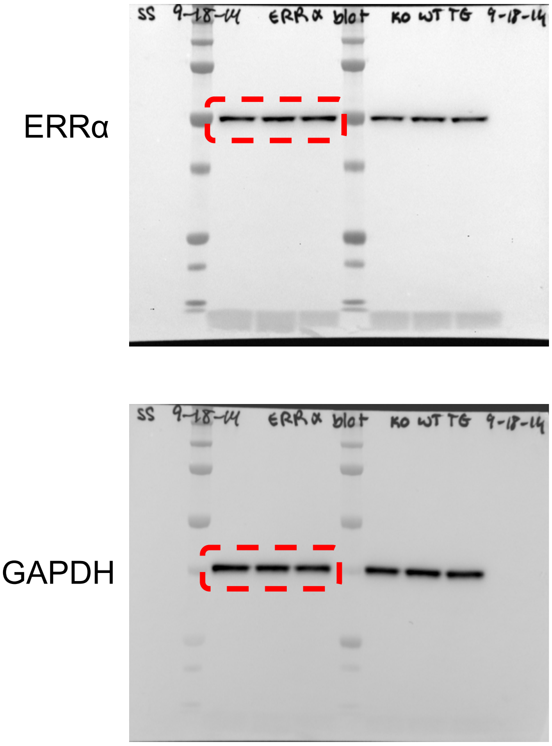

**Figure 6G**

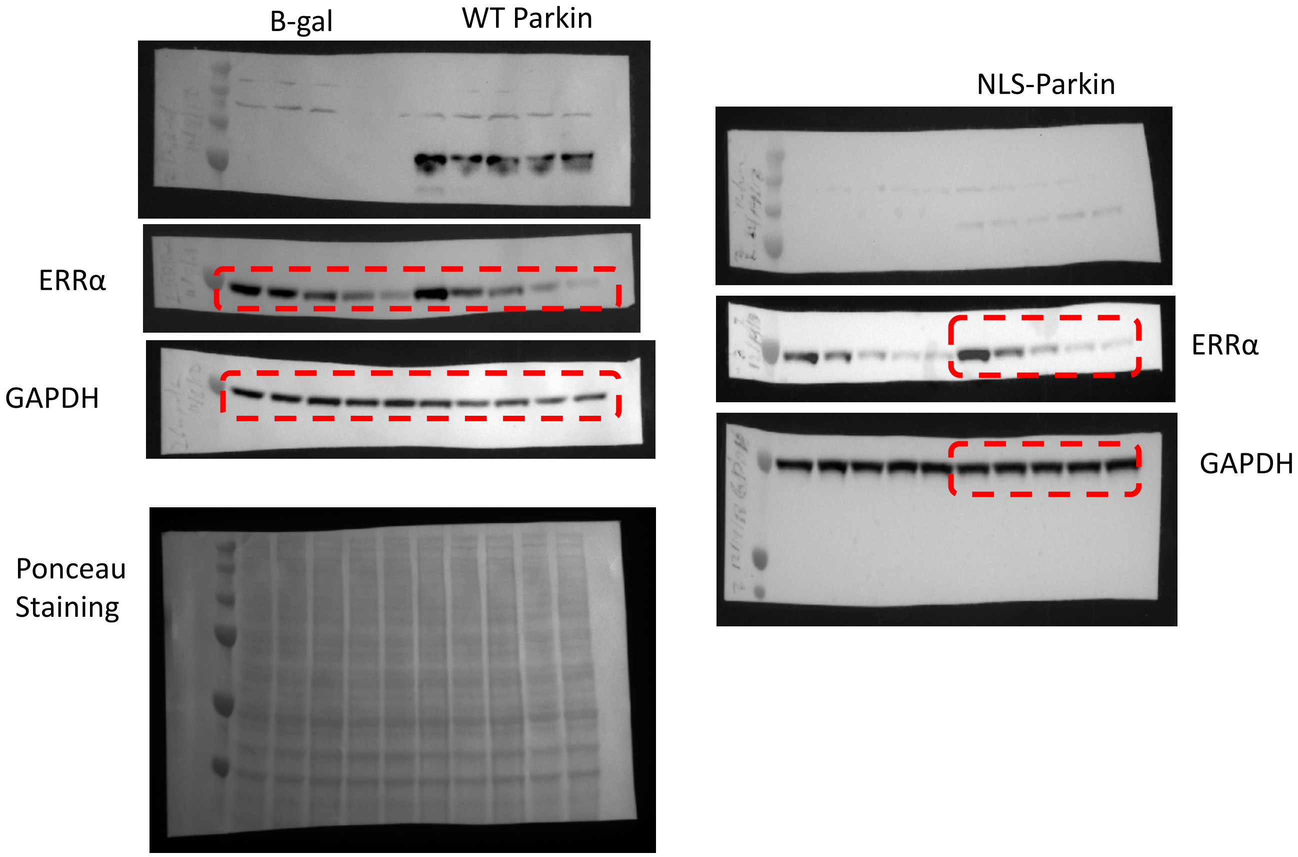
B-gal
WT Parkin
NLS-Parkin
ERRα
GAPDH
ERRα
GAPDH
Ponceau
Staining

**Supplemental Figure S2C**

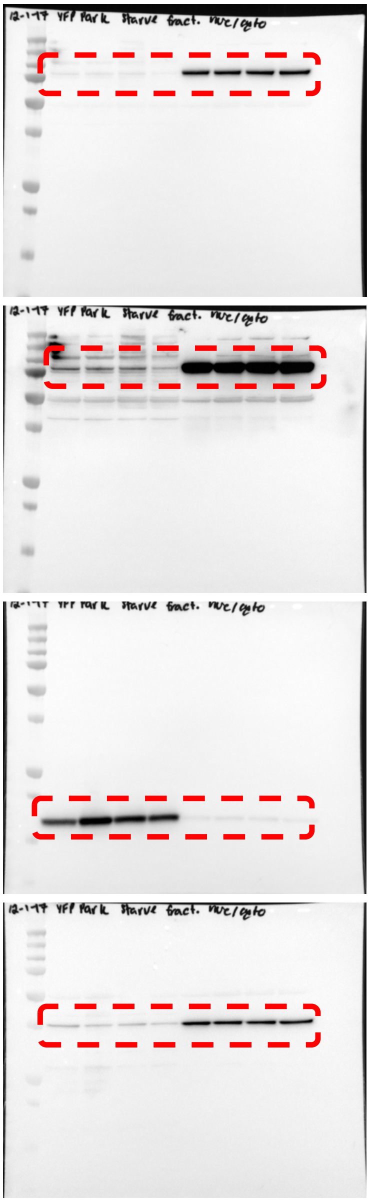

Parkin (short)

Parkin (long)

Histone H3

GAPDH

**Supplemental Figure S5A**

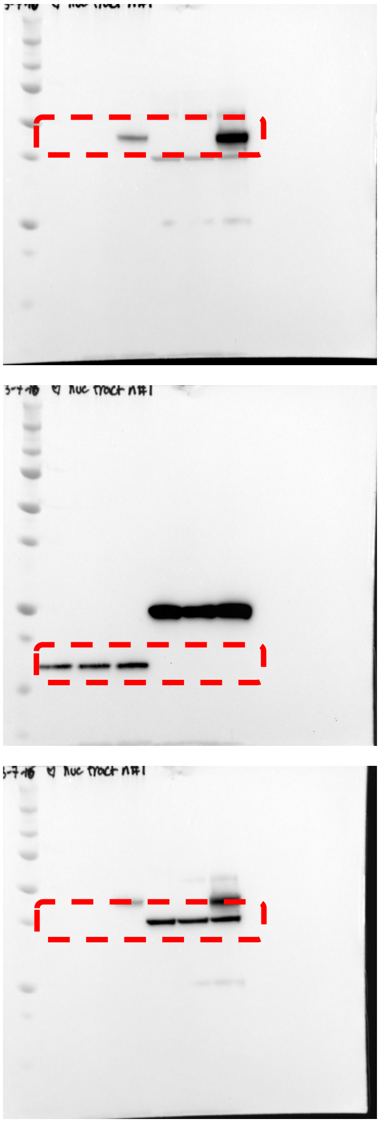

Parkin

Histone H3

GAPDH

**Supplemental Figure S5B**

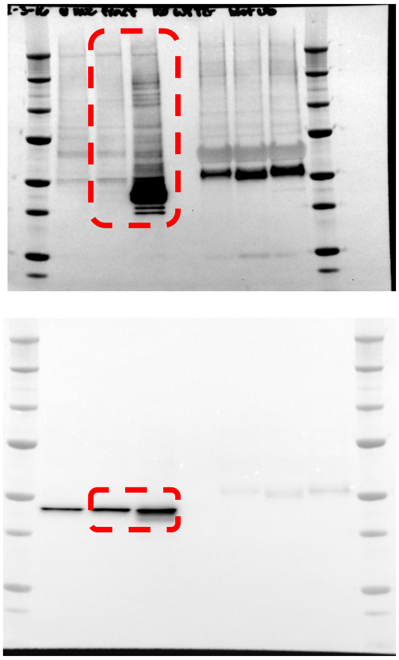

Ubiquitin

Lamin A/C

**Supplemental Figure S5D**

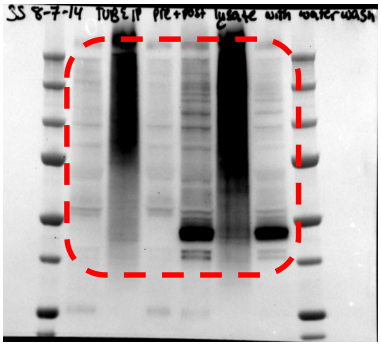


Ubiquitin
